# DNA metabarcoding analyses reveal fine-scale microbiome structures on Western Canadian bat wings

**DOI:** 10.1128/spectrum.00376-24

**Published:** 2024-10-22

**Authors:** Chadabhorn Insuk, Naowarat Cheeptham, Cori Lausen, Jianping Xu

**Affiliations:** 1 Department of Biology, Faculty of Science, McMaster University, Hamilton, Ontario, Canada; 2 Department of Biological Sciences, Faculty of Science, Thompson Rivers University, Kamloops, British Columbia, Canada; 3 Wildlife Conservation Society Canada, Kaslo, British Columbia, Canada; Brigham Young University, Provo, Utah, USA

**Keywords:** bat, DNA metabarcoding, amplicon sequencing, 16S rRNA, internal transcribed spacer (ITS), bat wing microbiome, white-nose syndrome (WNS)

## Abstract

Healthy wings are vital for the survival and reproduction of bats, and wing microbiome is a key component of bat wing health. However, relatively little is known about the wing microbiome of bats in western Canada where the white nose syndrome has become an increasing threat. Here, we used DNA metabarcoding to investigate the bacterial and fungal communities on the wings of three bat species: the big brown bat (*Eptesicus fuscus*), the Yuma myotis (*Myotis yumanensis*), and the little brown myotis (*M. lucifugus*) from four field sites in Lillooet, British Columbia, Canada. The bacterial 16S rRNA metabarcoding revealed a total of 4,167 amplicon sequence variants (ASVs) belonging to 27 phyla, 639 genera, and 533 known and 2,423 unknown species. The wing bacteria were dominated by phyla Proteobacteria, Firmicutes, Bacteroides, and Actinobacteria, and the most common genera were *Delftia*, *Bordetella*, *Sphingomonas*, *Phyllobacterium*, *Bradyrhizobium*, *Pseudomonas*, and *Corynebacterium*. The fungal internal transcribed spacer (ITS) metabarcoding revealed a total of 11,722 ASVs belonging to 16 phyla, 806 genera, and 1,420 known and 10,302 unknown species. The wing fungi were dominated by phyla Ascomycota, Basidiomycota, and Motierellomycota, and the most common genera were *Cladosporium*, *Aspergillus*, and *Mycosphaerella*. Principal coordinates analysis showed that both bat species and field sites contributed variably to the diversity and distribution of bacterial and fungal communities on bat wings. Interestingly, both positive and negative correlations were found in their relative abundances among several groups of microbial taxa. We discuss the implications of our results for bat health, including the management of *P. destructans* infection and white-nose syndrome spread.

**IMPORTANCE:**

Microbiomes play important roles in host health. White-nose syndrome (WNS), a fungal infection of bat wings and muzzles, has threatened bat populations across North America since 2006. Recent research suggest that the skin microbiome of bats may play a significant role in bat's susceptibility to WNS. However, relatively little is known about the skin microbiome composition and function in bats in Western Canada, a region with a high diversity of bats, but WNS has yet to be a major issue. Here, we revealed high bacterial and fungal diversities on the skin of three common bat species in Lillooet, British Columbia, including several highly prevalent microbial species that have been rarely reported in other regions. Our analyses showed fine-scale structures of bat wing microbiome based on local sites and bat species. The knowledge obtained from WNS-naïve bat populations in this study may help develop mitigation and management strategies against WNS.

## INTRODUCTION

Microbiome refers to the community of microorganisms that can form commensal, mutualistic, or pathogenic relationships with host species (1). Microbiomes are integral components of diverse ecosystems and are increasingly recognized for their roles in maintaining the health of hosts (2, 3). In animals, the skin is among the first lines of defense against pathogen invasion and often carries symbiotic microbiota that can influence host health (4). For example, the skin microbiome can produce antimicrobial molecules to inhibit the colonization of other harmful microorganisms and prime the innate and adaptive cutaneous immune systems of the hosts (5).

DNA metabarcoding is a tool that can provide rapid assessment of microbial diversity by using DNA sequencing to identify multiple taxa in a sample simultaneously (6). Such microbial inspection can have widespread applications, including for surveying distributions of pathogens. For example, Osman et al. (7) used DNA metabarcoding to study amphibian skin microbiome and monitor *Batrachochytrium dendrobatidis* (*Bd*), a pathogenic chytrid fungus, which has decimated more than 500 amphibian species worldwide (8, 9). Indeed, patterns of microbial diversity were associated with *Bd* infection, likely due to microbiome-mediated effects on *Bd* either directly or indirectly (10). Similarly, white-nose syndrome (WNS), caused by the fungus *Pseudogymnoascus destructans* (*Pd*), continues to threaten many species of bats in North America as identified by cutaneous infection of muzzles and wings, and associated mass mortality (11). Likewise, DNA metabarcoding can provide key information about the distribution of *Pd/*WNS and corresponding microbiome structure of bat skin in connection with WNS.

Bats are an ecologically and economically important group of species in natural ecosystems across the globe. They are the only true flying mammal, most of them have long life expectancy. They reproduce slowly (12) and are physiologically complex in their use of daily or seasonal torpor (including hibernation in many species [13]). Bats have unique immune systems that allow them to carry a wide range of viruses, some of which have mutated to become zoonotic pathogens (14). In North America where bats have not been associated with recent infectious disease in humans, bats provide significant services to agriculture and forestry industries and ultimately to humans through their significant consumption of insects, many of which are pests on agricultural crops or human disease-vectors (15). However, the North American bat populations are increasingly threatened by WNS. The ascomycete pathogen *Pd* has killed millions of bats since its first discovery in New York state in 2006 (16). Interestingly, susceptibility to *Pd* infection and disease severity of *Pd*-infected bats vary among species. For example, significant mortality associated with WNS has been documented in little brown myotis (*Myotis lucifugus*), northern long-eared bat (*My. septentrionalis*), and tricolored bat (*Perimyotis subflavus*), while other species (e.g., big brown bat [*Eptesicus fuscus*], Indiana bat [*My. sodalis*], and eastern small-footed bat [*My. leibii*]) show infrequently *Pd* infection with no or limited disease symptoms (17). Currently, WNS has been confirmed in 12 bat species in North America, including three endangered and one proposed endangered species in the US (18) and two endangered species in Canada (19). At present, the reason for the variations in susceptibility to WNS remains largely unknown (20). Researchers hypothesize that multiple factors determine WNS susceptibility among bats, including host immune system, body size, and body temperature; environmental temperature; geological structure of hibernacula; migratory distance; hibernation strategy, habitat; and skin microbiome (21–24).

Due to the importance of bats in environmental, economic, and human health aspects (25), it is critical to understand the factors that contribute to susceptibility differences in WNS among bats. Several studies have investigated the skin microbiome of North American bats and how skin microbiome may contribute to their WNS susceptibility differences, using culture-based assays (26, 27), metagenomics-based assays (28, 29), or a combination of these two approaches (30). These studies have revealed varied components of bat skin microbiome, with some fungi being transient, reflecting immediate environmental microbial community, and others being commensals that establish long-term presence on wings. Fungal and bacterial microbiomes varied with factors including geography, bat species, ecology (28–30), environmental temperature, and host body condition (31), but with no clear evidence of phylogenetic effects of bats (29). Several studies have found that the skin microbiome of bats infected with *Pd* have overall reduced microbial diversity than bats not infected with this fungus (32, 33), and in some cases, changes in bacterial microbiome with greater relative abundance of anti-fungal microbes have been documented (32, 33). For example, several bacterial genera isolated from bat wings have shown antifungal activities, such as *Enterococcus*, *Burkholderia*, *Flavobacterium*, *Pseudomonas*, *Corynebacterium*, and *Rhodococcus* (34). Indeed, several *Pseudomonas* bacteria isolated from bat skin exhibit high potential to produce antifungal metabolites against *Pd* (26, 27). Together, these studies suggest the potential of modulating the bat wing microbiome to enhance their resistance against *Pd* infections.

In Canada, while DNA metabarcoding-based microbiome studies have been conducted on bats from the eastern and central provinces of Manitoba, Quebec, and Ontario (23), there is no available information from British Columbia (BC), the province with the highest diversity of bats in Canada and where bats likely exert broader impacts on local ecology and environments (35). Due to geographic and climatic heterogeneity across Canada, the distribution of Canadian bats is also highly heterogeneous. Within BC, the western-most province in Canada where the distributions of bats are very patchy, there are diverse ecosystems that differ greatly in geography and climate. High diversity of bats and their heterogeneous distributions in BC may add another complexity to the skin microbiome of BC bats. For example, differences in interspecific interactions and sociality among BC bats could contribute to their distinctive microbiome structures that are different from those of eastern and central Canadian bats. In addition, the analyzed bat samples in individual studies have been highly heterogenous, often from broad geographic regions and ecological niches, and spanning multiple months and seasons. Such spatial and temporal heterogeneity could confound interpretations of the observed variations in bat skin microbiome, making our current understanding of bat microbiomes from eastern/central Canada not applicable to those in western Canada. Together, while the wing microbiome of bats in many parts of the globe has been analyzed, including in eastern North America where WNS is prevalent, relatively little is known about the wing microbiota of bats in western Canada where WNS has yet to spread widely (18, 24).

In this study, to minimize confounding factors in interpreting variations in bat wing microbiome, we investigated fine-scale geographic structures of the wing microbiome for three common bat species in Lillooet, BC, the area where WNS had not yet been reported as of July 2024. The three bat species analyzed here are: the big brown bat (*Eptesicus fuscus*; EPFU), the little brown myotis (*Myotis lucifugus*; MYLU), and the Yuma myotis (*Myotis yumanensis*; MYYU). In eastern North America, MYLU is highly susceptible to WNS (36), while EPFU is less susceptible to WNS (37, 23). MYYU is found only in western North America, and its susceptibility to WNS is currently unknown. In western North America, MYLU and MYYU distributions overlap with each other, and they commonly co-inhabit the same roosts. The Lillooet region is in interior BC, relatively isolated by big mountains from lowland BC (including Vancouver) where anthropogenic influences are more pervasive. Lillooet contains a diversity of habitats and supports a large number of bat species (38). We hypothesize bat wing microbiome differences exist within and between bats in our sampled region. In addition, we hypothesize that local habitat differences within the Lillooet area will contribute to microbial differences among their wing microbiomes, such that similar species’ ecologies will produce similar wing microbiomes. Specifically, MYLU and MYYU have similar foraging and roosting behaviors (38), we speculated that they should have more similar microbial communities to each other than either of them is to those of the big brown bat. Also, though no *Pd* was detected in our samples (see Results), we speculate that abundances of some taxa with reported anti-*Pd* activity will likely be negatively correlated with the relative abundances of fungi in the genus *Pseudogymnoascus*. To reduce potential confounding factors of geographical and temporal effects, all bat samples analyzed in this study were collected in Lillooet at around the same time (within 5 days). The wing microbial communities were analyzed using DNA metabarcode sequencing for both bacteria and fungi.

## MATERIALS AND METHODS

### Sample collection

**Fig 1:**
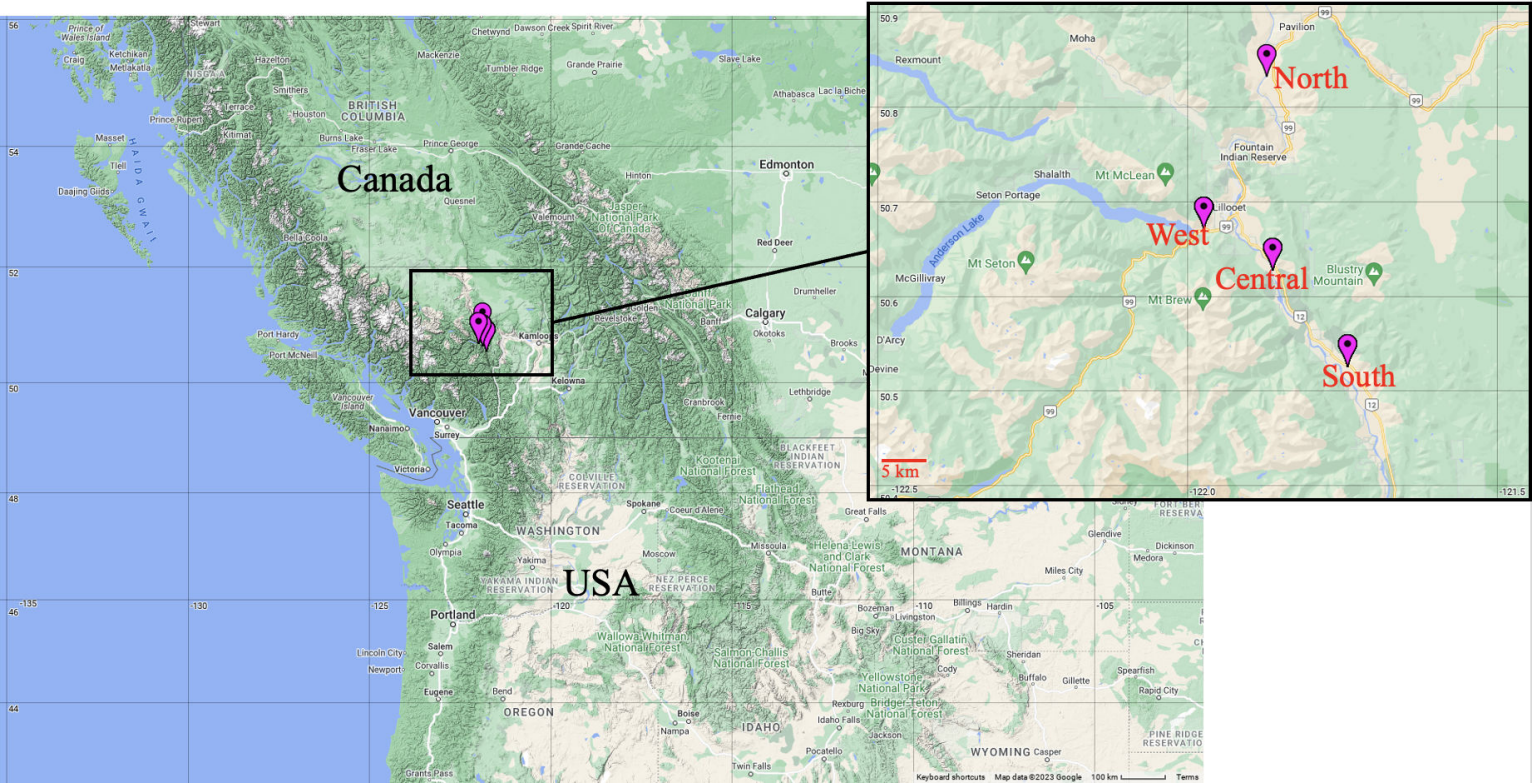
Sampling sites around Lillooet, BC, Canada. Map was generated by Map Maker online software (https://maps.co/gis/).

Sample collection took place at four field sites in Lillooet, British Columbia, Canada (Fig. 1), in late July 2022 (summer) under the permit of Cori Lausen (permit number MRCB20-598305, WCS Canada): North Lillooet site, South Lillooet site, Central Lillooet site, and West Lillooet site. The four field sites differed in geography and vegetation. The North Lillooet site was an open dry grassland adjacent to but >200 m above the Fraser River, touting rocky outcrops and limited human activity. The remaining three sites had frequent human activities in the immediate area where bats were captured. The Central Lillooet site was semi-forested grasslands near rocky outcrops, and offered artificial fishponds where bats drank and foraged; this ranch also had several bat boxes as artificial roosts occupied by big brown bats. However, there was no bat in the bat boxes during our capture. The South Lillooet site was an open grassland with few trees and a large dug-out of water where bats were captured as they came to drink or forage; this area was within 2 km of rocky outcrops and an abandoned mine. In contrast, the West Lillooet site was in a valley, surrounded by mountain forests, rugged rocky terrain, and close to a large body of water (Cayoose Creek and Seton Lake). We used mistnets to capture all three bat species Yuma myotis (MYYU), little brown myotis (MYLU), and big brown bats (EPFU).

Soon (within approximately 10 min) after each bat was caught, it was gently taken out of the mistnet and placed into a clean cloth bag. All the bags were cleaned, washed, and dried before their use. In addition, each bag was used at most only once each night. A new pair of sterile nitrile gloves were used to handle each bat and changed between bats to prevent cross contamination. Face masks were always worn by humans to minimize any possible exchange of microbes through respiratory droplets between humans and bats, as per handling guidance for bats (39). In addition, clean clothes were worn on each day to prevent microbial contamination among sampling sites, and all tools for bat capture and sampling were disinfected with 70% ethanol after each day’s work. Bats were identified to species and sex based on their morphological features and the frequency of echolocation calls by bat specialists (Cori Lausen and Heather Gates of Wildlife Conservation Society Canada). For each captured bat, a sterile cotton swab (Puritan) was moistened with sterile distilled water and rolled on both the dorsal and ventral sides of both wings, swabbing an area approximately 30 cm^2^. We placed each swab into a sterile Eppendorf tube and stored the tubes in −20°C freezer within 2–8 h after sampling until DNA metabarcode sequencing.

### Sample preparation and sequencing

Sample preparation for sequencing was done in a biosafety cabinet. One thousand microliters of filtered sterile distilled water was added into each Eppendorf tube that contained a swab. The tube was then vortexed for 30 s to dislodge microbial cells from the swab, and 500 µL of the suspension was transferred to a new sterile Eppendorf tube. The new tubes were sent on ice to Metagenom Bio Inc. (Waterloo, Ontario) for metabarcode sequencing following their standard protocols for bacterial and fungal community analyses. Briefly, genomic DNA was extracted with the Sox DNA Isolation Kit (Metagenom Bio Inc.) from each sample according to the supplier’s recommendation. PCR was set in triplicates for each sample (25 µL each). Negative control with sterilized distilled water was included to check for potential microbial contamination in PCR and sequencing reagents. Each reaction mixture contained 2.5 µL of 10× standard Taq buffer, 0.5 µL of 10 mM dNTP, 0.25 µL of BSA (20 mg/mL), 5.0 µL of 1 µM forward primer, 5.0 µL of 1 µM reverse primer, 5.0 µL DNA, 0.2 µL of Taq DNA polymerase (5 u/µL), and 6.55 µL of PCR water. DNA was denatured at 95°C for 5 min, followed by 35 cycles of 95°C for 30 s, 30°C for 30 s, and 72°C for 50 s and then extended at 72°C for 10 min. For bacterial community analyses, the 515FB/806RB universal 16S V4 rRNA primers (515FB: 5′-GTGYCAGCMGCCGCGGTAA, 806RB: 5′-GGACTACNVGGGTWTCTAAT) (40) were used. For fungal community analyses, the BITS/B58S3 universal fungal ITS primers (BITS: 5′-ACCTGCGGARGGATCA-3′, B58S3: 5′-GAGATCCRTTGYTRAAAGTT-3′) (41) were used.

The triplicate PCR products for each wing swab sample were pooled, resolved with 2% TAE agarose gel, gel purified, and quantified using Qubit dsDNA HS Assay Kit (ThermoFisher Scientific Inc.). The purified product from each sample was ligated to unique adaptor sequences, and the library DNA was sequenced with MiSeq Reagent Kit v2 (paired end 2 × 250 bp). FASTQ files were generated for taxonomic and sequence analyses.

### Bioinformatic processing of the sequences

Demultiplexed sequences were processed using *cutadapt* for primer removal (42). *DADA*2 v.1.22 was used for sample inference and denoising to assign taxonomy to individual sequences because of its high accuracy and high resolution for inferring amplicon sequence variants (ASVs) with even one or two nucleotide differences (43). Paired-end reads were truncated at decreasing quality scores and assembled. After quality filtering and taxonomic assignment, an abundance table was constructed. This abundance table records the number of sequence reads for each ASV. Taxonomy was assigned to representative sequences using naive-Bayes classifier trained against a reference database (16S rRNA: SILVA release 138 at 99% [44]; ITS: UNITEdb ver. eight dynamic [45]). The primers used in marker analysis could amplify a small number of non-target sequences, such as organelles (chloroplast and mitochondria) and sequencing errors, including chimeras. These were removed from processed data before any statistical analysis was performed. One *Pseudomonas* sequence from the 16S data was dropped, as it was seen in the negative controls. This sequence was the only one that showed up in most negative controls from our samples, thus it was excluded from all downstream analysis. ASV table, taxonomy table, and metadata were combined into a *phyloseq* object using the *phyloseq* package (46) v1.42.0. All downstream statistical analyses and visualizations were performed in R v4.2.2 (47), as described below.

### Statistical analyses and data visualization

Statistical analyses and data visualization were performed in R using packages including *phyloseq* (46), *tidyverse* (48), *dplyr* (49), *vegan* (50), *metagMisc* (51), and *microbiom*e (52). Visualization was done using *ggplot2* (53), *MicEco* (54), *circlize* (55), *RColorBrewer* (56), *ComplexHeatmap* (55), and *microViz* (57) packages. Alpha diversity and beta diversities were calculated from rarefied sequence reads. Rarefaction was performed by subsampling all samples to the sequence count of the smallest library of 2,000 reads for both the 16S rRNA and the ITS data sets (100 iterations). R *vegan* package was used to calculate Alpha diversity and richness, including observed ASVs, Chao1, abundance-based coverage estimator (ACE), Simpson index, inverse Simpson index (InvSimpson), Shannon diversity index, and Fisher’s index. We applied one-way analysis of variance (ANOVA) to test for differences on every pair of alpha diversity among the four field sites for each of the three bat species. *P*-values from ANOVA were corrected by Benjamini–Hochberg (BH) false discovery rate (FDR) correction to control for type I error or false positives in all rejected null hypothesis. BH correction test was chosen because it is less conservative than Bonferroni method and is commonly used in *P*-value adjustment method for microbiome (58). Diagnostic plots of residuals and quantile–quantile normality plot did not violate the assumption of linear model and homoscedasticity. No outliers over Cook’s distance indicated no big influence of outliers on data (File S1). Beta diversity matrix was calculated using the Bray–Curtis dissimilarity function and projected onto two-dimensional space by principal coordinates analysis (PCoA). Permutational multivariate analysis (PERMANOVA) was performed using *adonis2* function in *vegan* package with statistical significance derived using 1,000 permutations. Pairwise PERMANOVA test was used to compare difference between groups, with the *P*-values adjusted based on BH-FDR correction. In addition, log-transformed relative abundances were used to assess the relationship between bacterial and fungal diversity/reads among samples using Pearson’s correlation coefficient (rho).

In order to compare our results with the report from eastern Canada, we retrieved raw sequence files of little brown bat skin microbiota published by (33) (from figshare (Raw sequence files: https://figshare.com/s/623a1e47b4bed20459a7; Metadata: https://figshare.com/s/74d9497a792f9c0c76df) and performed 16S rRNA metabarcoding analysis through our pipeline including adapter removal and classifying reads by our classifier as mentioned above. Only bacterial diversity was obtained and analyzed in that study (33). We compared taxonomic assignment and abundance profile of microbiome with our data to control for bias when making comparison.

### Real-time PCR (qPCR) procedure for *Pseudogymnoascus destructans* (*Pd*) detection

*Pd* strain US-15 was grown as pure culture on Sabouraud dextrose agar and incubated at 14°C for 14 days or until sporulated. Spores were harvested in a level two biosafety cabinet using the method described by (59). Briefly, conidia harvesting solution (CHS, 0.05% Tween 80, 0.9% NaCl) was added to submerge the agar surface, then the surface was scraped by sterilized loop to dislodge conidia. Spores were filtered through sterile glass wool packed in a sterile glass funnel to a sterile 15 mL falcon tube. Conidia were washed and stored in phosphate buffered saline (PBS, pH 7.0) at 4°C until needed. Spore numbers were counted under a hemocytometer and adjusted for qPCR standards. Spore concentrations of 10^4^, 10^5^, 10^6^, and 10^7^ spores/mL were used as positive controls. Dilutions of positive controls mentioned above were used to create a standard curve and calculate for R^2^ value (R^2^ = 0.9981). qPCR procedure was adapted from (60). Primers and probe used were as follows: forward primer: 5′-TGC CTC TCC GCC ATT AGT-3′; reverse primer: 5′-ACC ACC GGC TCG CTA GGT A-3′; and probe /56-FAM/CG TTA CAG T/ZEN/T GCT CGG GCT GCC /3IABkFQ/ (Integrated DNA Technologies). Each 20 µL qPCR reaction contained 10 µL 2× Luna probe qPCR master mix (New England Biolabs), 0.5 µL of each 10 µM PCR primer solution, 0.25 µL 10 mM probe, 2.75 µL UltraPure water (Invitrogen), and 6 µL template DNA taken from a tube of dry swab hydrated in Tris-EDTA buffer (pH 8.0). Four positive controls of *Pd* spore concentrations and a no-template control were included in each 96-well assay plate. Negative control used sterilized PBS buffer in place of *Pd* spore solutions. qPCR cycling condition was 95°C for 3 min, 40 cycles of 95°C for 5 s, and 60°C for 30 s. qPCR was set in triplicates, and standard practice was performed to prevent contamination. qPCR was run in a CFX Opus 96 qPCR machine (Bio-Rad) using SYBR/FAM channel. Then, the results were analyzed in Bio-Rad CFX Maestro software. Any reaction that crossed the threshold baseline within 40 cycles was considered positive (51).

## RESULTS

### Bat capture

Between July 24 and July 28, 2022, at four field sites around Lillooet, BC, we captured a total of 76 bats of the three target species: 40 EPFU, 26 MYYU, and 10 MYLU (Table 1). At the North Lillooet site, we only captured EPFU. At the South Lillooet and Central Lillooet sites, we captured two of the three bat species: EPFU and MYYU. At the West Lillooet site, bats belonging to all three species (EPFU, MYYU, MYLU) were captured. None of the bats showed any signs of sickness and all were capable of flying after wing swabbing and release.

**TABLE 1:** Bats captured and analyzed for this study

Date of capture	Site name	No. of bats and sex		
		EPFU^a^	MYYU^b^	MYLU^c^
July 24, 2022	North Lillooet	2 (males)	0	0
July 25, 2022	South Lillooet	15 (3 males, 12 females)	3 (1 male, 2 females)	0
July 26, 2022	Central Lillooet	16 (1 male, 15 females)	1 (female)	0
July 28, 2022	West Lillooet	7 (males)	22 (5 males, 17 females)	10 (females)

^*a*^ EPFU, *Eptesicus fuscus*.

^*b*^ MYYU, *Myotis yumanensis*.

^*c*^ MYLU, *Myotis lucifugus*.

### Wing bacterial community

#### Bacterial community compositions and relationships

**Fig 2:**
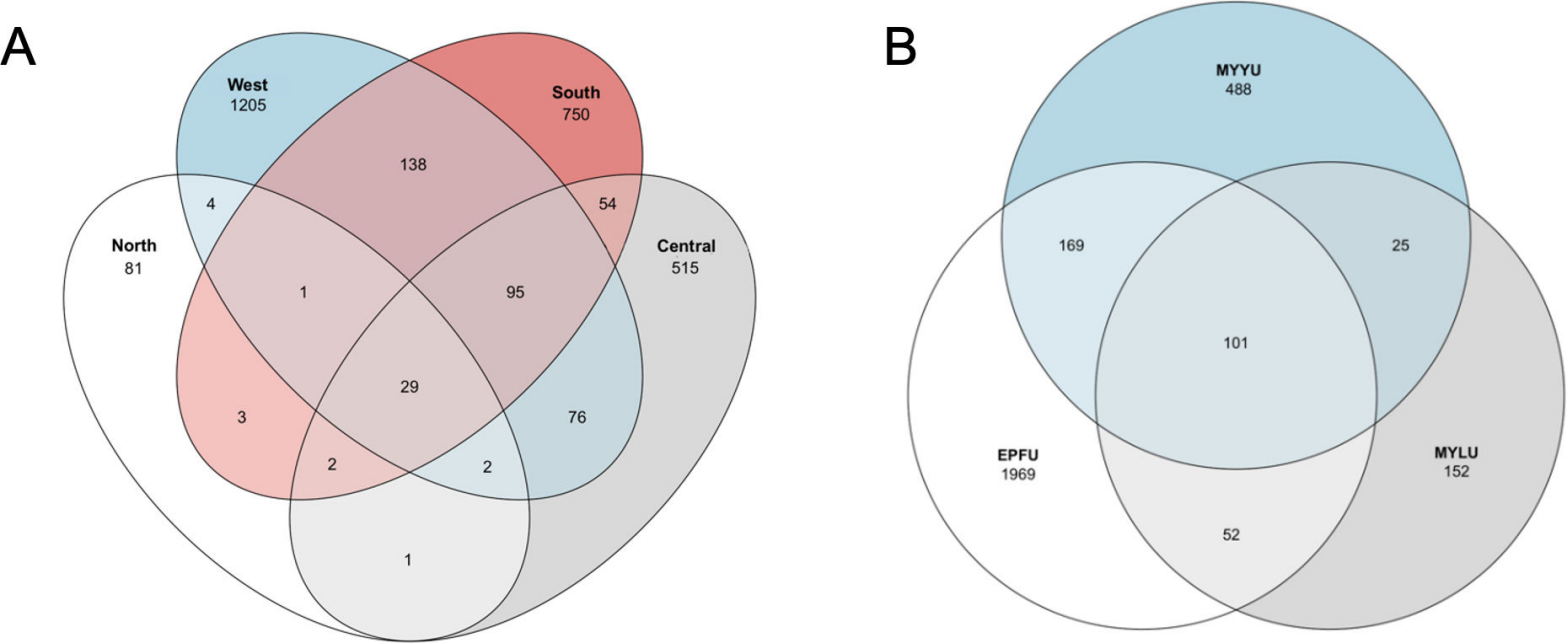
Distribution of bacterial species/OTUs among fields sites (**A**) and host bat species (**B**).

Bat wing microbiome composition was investigated using high throughput DNA metabarcoding and bioinformatic analyses. From 16S rRNA analysis, after filtering, the total number of reads from all 76 bat wing swab samples was 3,237,345, with an average of 42,597 reads per sample (a range of 664 to 108,379 reads). The average length of reads was 253 bp. These 3,237,345 reads belonged to 4,167 ASVs, and these ASVs were clustered into 27 phyla, 60 classes, 155 orders, 272 families, 639 genera, and 533 known species, and 2,423 unknown species. The distributions of the total 2,956 bacterial species among the four field sites and three bat species are shown in Fig. 2. Overall, the West Lillooet site had the highest number of bacterial species, followed by the South, Central, and North Lillooet sites. The four sites shared 29 bacterial species. Among the three bat species, EPFU had the highest number of bacterial species, followed by MYYU and MYLU. The three bat species shared 101 bacterial species on their wings (Fig. 2).

**Fig 3:**
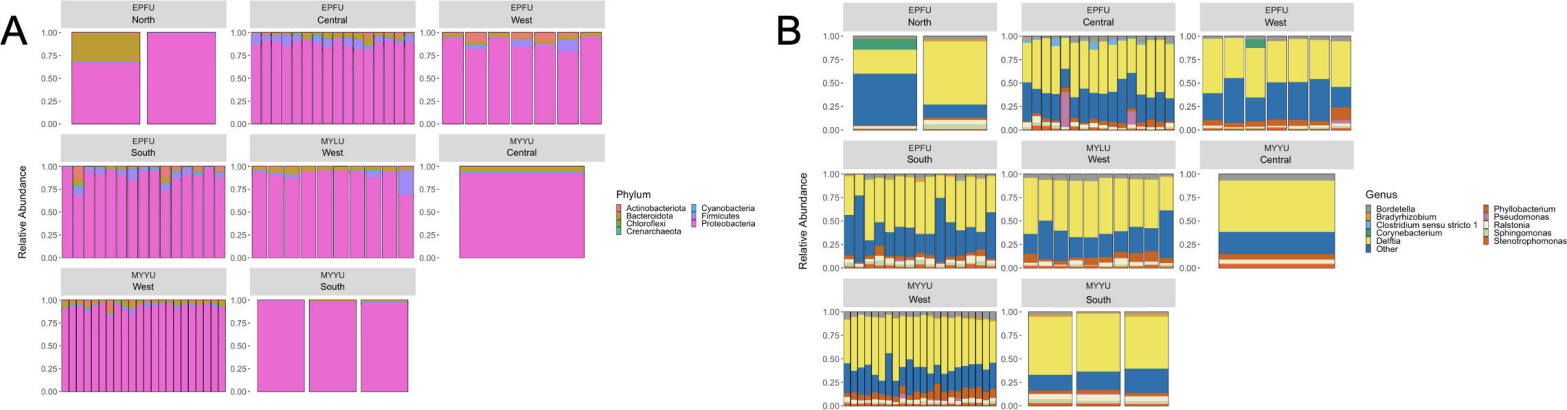
Relative abundance of 16S rRNA reads of bacteria at the phylum (**A**) and genus levels (**B**) for each of the 76 samples, by bat species and field site.

At the phylum level, the compositional bar plot of bacterial species with an abundance of >1% (inferred based on the total 16S rRNA data set) is shown in Fig. 3. The wing microbiome of Lillooet bats was primarily comprised of Proteobacteria, Bacteroidota, Firmicutes, and Actinobacteria (Fig. 3A). Proteobacteria comprised a vast majority of observed bacterial species at all four field sites and in all three bat species (Fig. 3A). Archaea species in the phylum Crenachaeota were also observed at all four sites and on all three bat species, but they were less than 1% in abundance in our samples. The top bacterial classes in Lillooet bat wing microbiome were Gammaproteobacteria, Alphaproteobacteria, Actinobacteria, Bacilli, Bacteroidia, Clostridia, Cyanobacteria, Rubrobacteria, and Thermoleoplilia.

The distribution of bacteria belonging to the overall top 10 genera on each bat is shown in Fig. 3B. At the genus level, *Delftia* was overall the most abundant and the most frequently detected bacterial genus on all individual bats across all four sites (100% of the samples; 22.28%–67.16% relative abundance) (Fig. 3B). Depending on location and bat species and individuals, other common genera were found, including *Bordetella* (100% of the samples; 0.46%–8.78% relative abundance)*, Sphingomonas* (100% of the samples; 0.08%–5.41% relative abundance)*, Phyllobacterium* (100% of the samples; 0.68%–17.17% relative abundance), *Bradyrhizobium* (77.63% of the samples; 0%–0.22% relative abundance)*, Pseudomonas* (76.31% of the samples; 0%–37.38% relative abundance)*,* and *Corynebacterium* (57.89% of the samples; 0%–9.19% relative abundance). *Pseudomonas* was found in high abundance especially from EPFU at the Central Lillooet site and the West Lillooet site. *Bordetella* and *Phyllobacterium* were found in high abundance across three of the four sites (except at the North Lillooet site).

**Fig 4:**
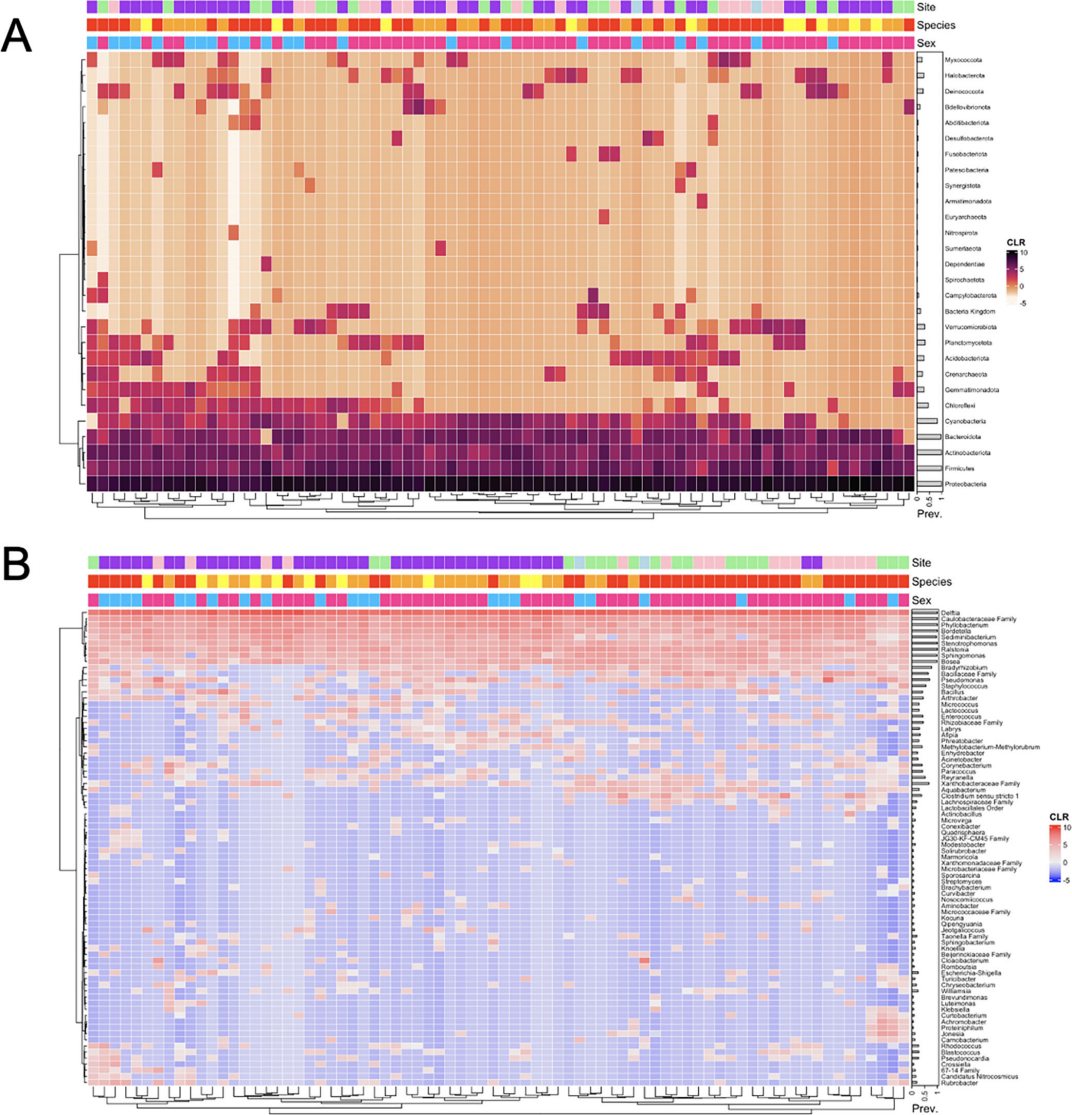
Heatmap showing relationships among bat wing microbiome and the relative abundances of bacteria at the phylum level (**A**) and genus level (**B**).Annotation bar color code: Top bar: Purple = West Lillooet; light pink = Central Lillooet; green -- South Lillooet; blue = North Lillooet. Middle bar: Red = EPFU; orange = MYYU; yellow = MYLU. Bottom bar: Pink = female; blue = male. Prev.: relative prevalence in the total sample; CLR: centered log-ratio of read counts in individual samples.

The similarities and differences of wing bacterial communities among the 76 bats are shown in Fig. 4 (4A at the phylum level and 4B at the genus level). Here, the only species with >1% relative abundance in the total sample was included. In this analysis, sequence read abundance values were transformed into centered log-ratio (CLR) and plotted using the Bray–Curtis distance to cluster the samples based on bacterial community composition. Samples with similar bacterial community composition were clustered near each other on the dendrogram. Overall, there was no evidence for exclusive clustering based on either bat species or field sites. However, highly similar bacterial communities were found among bats at the same site and/or belonging to the same species. For example, at the phylum level, three high-similarity clusters each containing four or more bats were found at the West Lillooet site, with each cluster containing microbiomes from two to three bat species. Two of these three clusters contained microbiomes from bats of both sexes. Similarly, there were several clusters of EPFU bats with highly similar bacterial communities at the phylum level, with some including bats from the same field site while others had bats from different field sites (Fig. 4A). Interestingly, a few small sex-based microbiome clusters involving different host bat species and/or from different field sites were also observed (Fig. 4A).

At the genus level, genera with >5% relative abundance in the total samples were included to show the relationships among the 76 samples (Fig. 4B). Similar to that observed at the phylum level, overall, there was no evidence for exclusive clustering based on either bat species or field sites. However, highly similar bacterial communities were found among bats at the same field site and/or belonging to the same bat species. Indeed, the overall field site- and host bat species-based clustering was more pronounced at the genus level than at the phylum level. For example, except the North Lillooet site where only two bats were sampled, each of the remaining three field sites had clusters of three or more samples with several clusters containing two or more bat species. Similar to that shown in Fig. 2B, the field site-specific microbiome clusters were often associated with site-specific bacterial genera. For example, at the South Lillooet site, the right-most cluster of samples in Fig. 4B contained genera that were rarely seen at other locations, including genera *Klebsiella*, *Curtobacterium*, *Achromobacter*, *Proteiniphilum*, *Jonesia*, and *Carnobacterium*. On the other hand, genus *Afipia* was commonly found on bats from the West Lillooet site. Interestingly, five EPFU bats (mostly male) from the West Lillooet site in the left bottom corner of Fig. 2B harbored several highly abundant bacterial genera, such as *Rhodococcus*, *Blastococcus*, *Pseudonocardia*, and/or *Crossiella*.

#### Fungal community compositions and relationships

**Fig 5:**
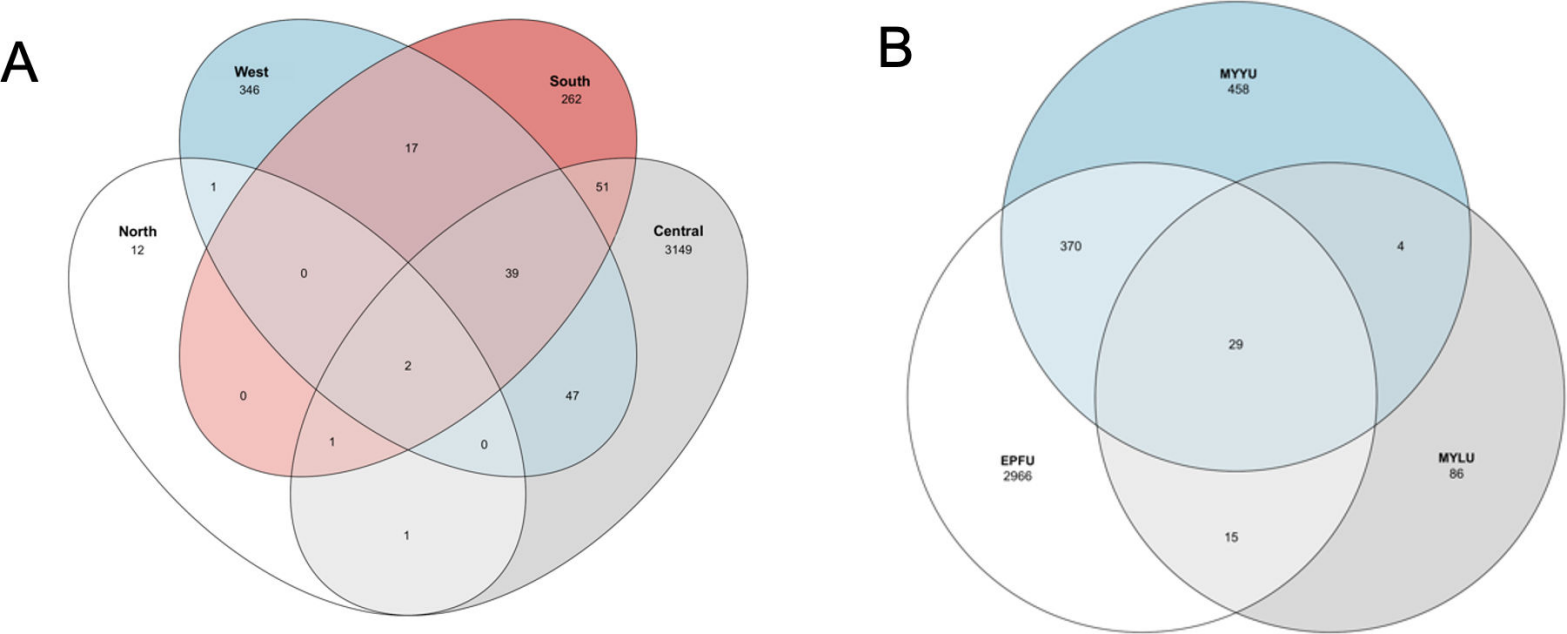
Distribution of fungal species/OTUs among fields sites (**A**) and host bat species (**B**).

The total number of ITS reads after filtering from all 76 samples was 3,094,855, with the average number of 40,722 reads per sample (a range of 313–250,584 reads). The average read length was 210 bp. These 3,094,855 reads were clustered into 16 phyla, 52 classes, 132 orders, 351 families, 806 genera, 1,420 known species, and 10,302 unknown species. Among all the sites, the Central Lillooet site had the highest number of fungal species, followed sequentially by the West Lillooet, the South Lillooet, and the North Lillooet sites. The four field sites shared only two fungal species. Among the three bat species, EPFU had the highest number of fungal species, followed by MYYU and MYLU. The three bat species shared 29 fungal species on their wings (Fig. 5).

**Fig 6:**
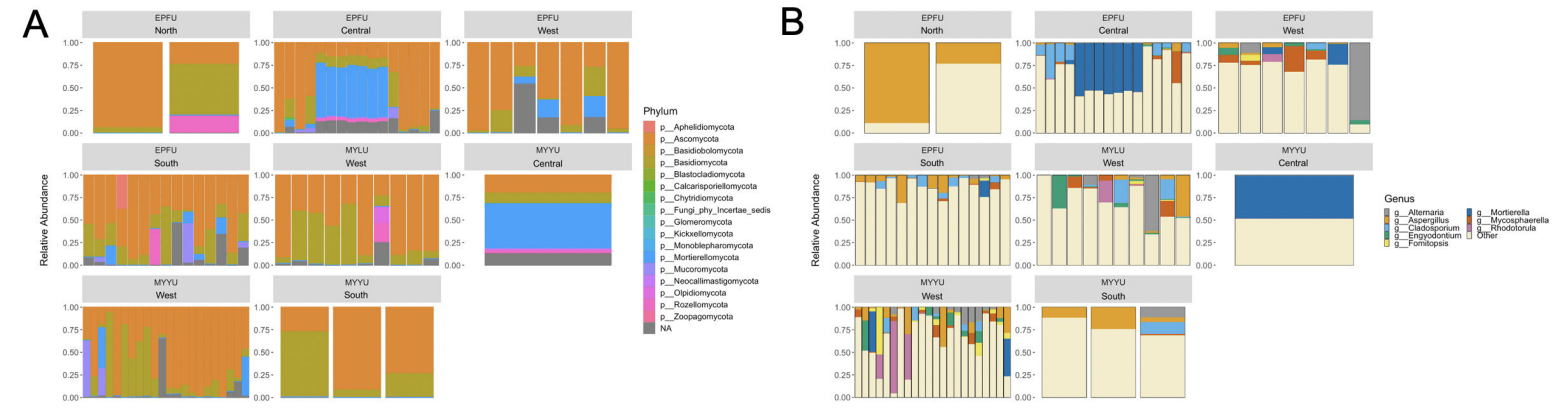
Relative abundance of ITS reads at the fungal phylum level (**A**) and genus level (**B**) for each of the 76 samples organized based on bat species and field sites.

At the phylum level, the wing mycobiomes of Lillooet bats were primarily comprised of Mortierellomycota, Ascomycota, Basidiomycota, and Rozellomycota. The compositional bar plot of fungal species with an abundance of >1% inferred based on ITS data set is shown in Fig. 6A. A few bats of both EPFU and MYLU contained relatively abundant reads in phyla Mucoromycotina and Zoopagomycota. Fig. 6B shows the relative abundance of fungi belonging to the overall top 10 genera on each bat. At the genus level, *Cladosporium* was overall the most abundant and the most predominant on almost all individual bats across all four field sites. Depending on location and bat species and individuals, other common fungal genera included: *Mortierella* (46% of the samples, 0%–57.44% relative abundance), *Aspergillus* (36.84% of the samples, 0%–9.88% relative abundance)*,* and *Rhodotorula* (14.47% of the samples, 0%–15.63% relative abundance). For example, on EPFU wings, *Aspergillus* was a common fungal genus; *Mortierella* was found in high abundance especially from EPFU and MYYU at the Central Lillooet site; and *Engyodontium* was common only at the West Lillooet site.

**Fig 7:**
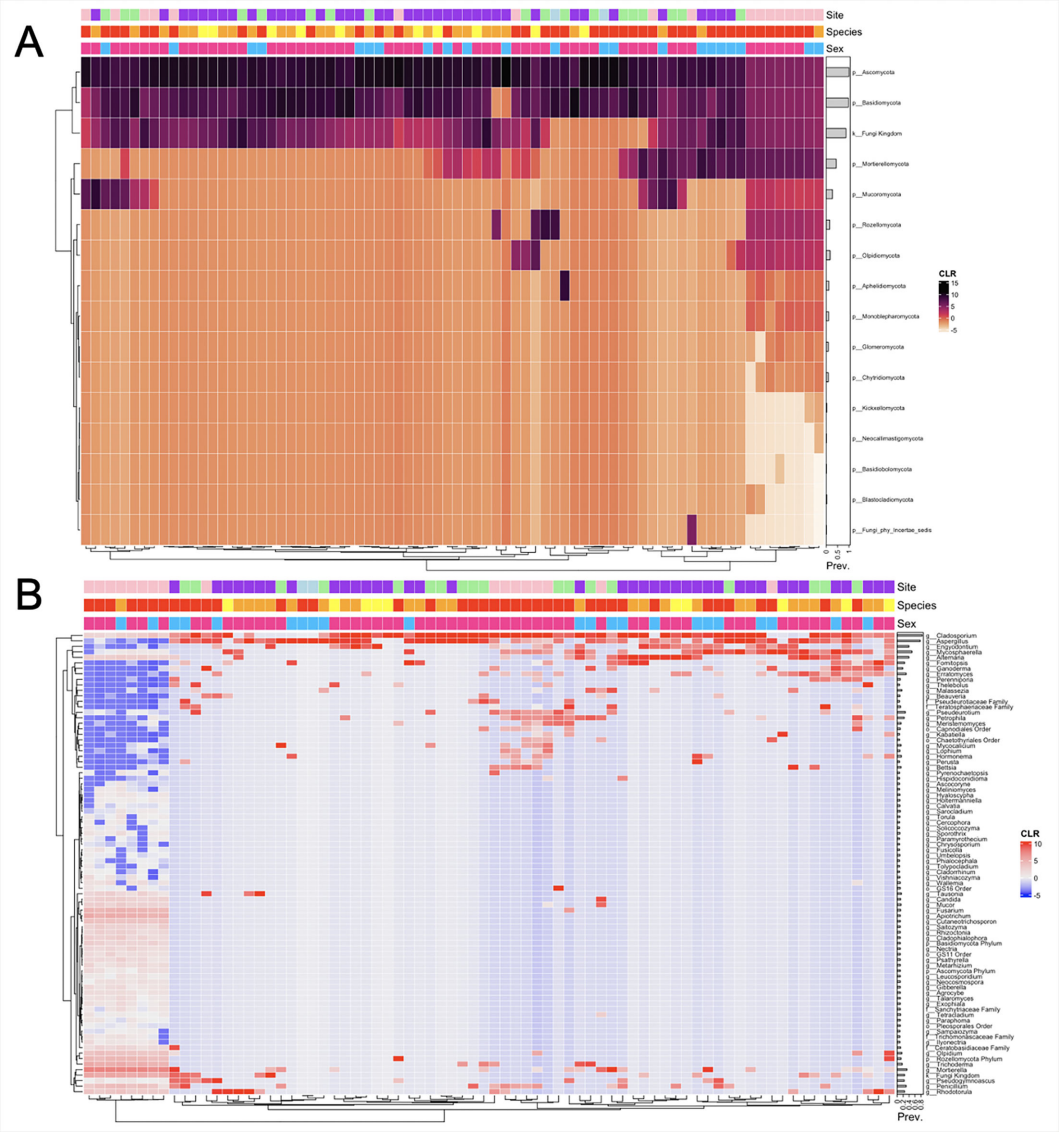
Heatmap showing relationships among bat wing microbiome and the relative abundances of fungi at the phylum level (**A**) and genus level (**B**).

Fig. 7 depicts the similarities among wing fungal communities of the 76 bats. Only phylum (Fig. 7A) and genus (Fig. 7B) at >1% and 5.5% relative abundances respectively in the total sample are shown in Fig. 7. The abundance values were transformed into centered log-ratio (CLR) and plotted using Bray–Curtis distance to cluster the samples based on fungal community similarities. Similar to the 16S rRNA data set, there was no evidence for exclusive clustering based on bat species or field sites. Nevertheless, several mycobiome clusters from the same field site and/or of the same bat species were found. For instance, at the phylum level, five high-similarity clusters each containing mycobiomes from three or more bats were found at the West Lillooet site, with each cluster containing two to three bat species. Two of these three clusters contained bats of both sexes (Fig. 7A). At the phylum level, mycobiomes of many EPFU bats from different field sites were clustered together in Fig. 7A. Host sex-based cluster was found (in the right-handed cluster of female EPFU) from the Central Lillooet site that harbored unique mycobiomes with highly abundant fungal phyla such as Mucoromycota, Rozellomycota, Olpidiomycota, Alphelidiomycota, and Monoblepharomycota. In addition, a few sex-based clusters involving different host species and/or from different sites were also observed (Fig. 7A).

Fig. 7B shows the relationships among the mycobiome samples at the genus level from genera that had >5.5% relative abundance. There was no evidence of exclusive clustering based on either bat species or field sites. The cluster of female EPFU bats from the Central Lillooet site showed highly similar fungal communities among bats at this site. However, bats from different field sites and/or belonging to different bat species also showed similar fungal communities. Overall, the clustering of mycobiome based on field site and host bat species was more prominent at the genus level than at the phylum level. At the field site level, the prominent clustering was found at the Central Lillooet site with two clusters of 6–8 bats. One of the two clusters included two species of bats: EPFU and MYYU. Field site-specific fungal genera were observed at the Central Lillooet site. At this site, a cluster of EPFU had a distinct pattern from the rest of the data set. This cluster comprised of low abundance of common genera from other clusters (*i.e.*, *Aspergillus*, *Alternaria*, *Engyodontium*, and *Mycosphaerella*), but comprised of high abundance of genera *Olpidium*, *Trichoderma*, *Mortieralla*, *Penicillium*, *Candida*, and *Rhodotorula*, which were not common in most of the other samples. The genus *Fomitopsis* (a shelf mushroom) was commonly found from the West Lillooet site. Interestingly, the genus *Pseudogymnoascus* was found on the wings of 21 bats distributed among all four field sites (27.63%). Among these 21 bats, 19 were EPFU, and the other two were MYYU. The bat with the highest abundance of *Pseudogymnoascus* was a big brown bat at the West Lillooet site where 12% of the ITS reads belonged to this genus. The white-nose syndrome causing pathogen, *Pd*, belongs to this genus. However, detailed sequence comparisons revealed that none of the *Pseudogymnoascus* ITS sequence reads were identical to *Pd*. In addition, qPCR analyses using *Pd*-specific primers yielded no amplification in any of the 76 samples.

### Community diversity analyses

#### Alpha diversity

##### 16S rRNA data set

For bacterial diversity comparisons among field sites and bat species, the 16S rRNA reads were normalized by rarefying at 2,000 reads per sample. Next, we calculated alpha diversity of all the four field sites and the three bat species (Table 2). We performed pairwise comparisons between the alpha diversity of bat species within the same field site. Here, the West Lillooet site was the only site that contained all three bat species and thus was the only site that we tested. Similarly, EPFU was the only species found at all four field sites and thus was the only bat species analyzed when conducting pairwise comparisons among the four sites. When we controlled for bat species by analyzing EPFU from all four sites, only Chao1 index showed significant difference between the Central and West Lillooet sites (*P*-value = 0.044). In contrast, ANOVA revealed that bat species contributed significantly to variations in alpha diversity indices at the West Lillooet site (one-way ANOVA with BH correction on bats from all sites, observed *P* = 0.0001, Chao1 *P* = 0.0004, ACE *P* = 0.0003, Shannon *P* = 0.0002, Simpson *P* = 0.02657, InvSimpson *P* = 0.0464, Fisher *P* = 0.0005). Specifically, the EPFU vs MYYU comparison showed significant differences for six of the seven indices (bats from all sites: observed = 0.00036, Chao1 = 0.0013, ACE = 0.0011, Shannon = 0.00043, Simpson = 0.03, InvSimpson = 0.06, Fisher = 0.0012). The EPFU vs MYLU comparison showed significant differences in five of alpha diversity indices (bats from all sites: observed = 0.00439, Chao1 = 0.0066, ACE = 0.0067, Shannon = 0.00890, Simpson = 0.20, InvSimpson = 0.20, Fisher = 0.0102). However, the MYYU vs MYLU comparison revealed no significant differences in the alpha diversity. Together, results from all three bat species at the West Lillooet site showed that MYYU and MYLU had similar alpha diversity, but both of them were significantly different from EPFU (*e.g.*, EPFU vs MYLU: observed = 1.2e-06; Shannon: 9.6e-05) (e.g., EPFU vs MYYU: observed = 4.4e-07; Shannon = 6.6e-05) (File S1).

**TABLE 2:** Bacterial species richness and alpha diversity indices based on 16S rRNA metabarcoding of bat wing microbiomes in Lillooet, BC, Canada

Index	Bat species^a^	Field site				
		North Lillooet	South Lillooet	Central Lillooet	West Lillooet	
Observed	EPFU	61.5 ± 44.55	83.33 ± 50.90	38.44 ± 72.01	87.86 ± 72.01	
	MYLU	NA	NA	NA	53.50 ± 27.87	
	MYYU	NA	45.67 ± 24.02	36	48.91 ± 25.21	
Chao1	EPFU	62.75 ± 46.31	79.06 ± 47.89	74.32 ± 46.95	118.89 ± 75.67	
	MYLU	NA	NA	NA	53.60 ± 32.44	
	MYYU	NA	44.58 ± 18.34	38	56.83 ± 27.49	
ACE	EPFU	64.05 ± 47.64	80.55 ± 49.03	75.52 ± 47.07	122.85 ± 77.83	
	MYLU	NA	NA	NA	53.30 ± 31.30	
	MYYU	NA	44.99 ± 18.37	42.10	57.38 ± 27.72	
Shannon	EPFU	2.26 ± 0.96	2.35 ± 0.59	2.28 ± 0.45	2.36 ± 0.57	
	MYLU	NA	NA	NA	2.03 ± 0.38	
	MYYU	NA	1.91 ± 0.65	1.46	1.97 ± 0.27	
Simpson	EPFU	0.72 ± 0.23	0.72 ± 0.11	0.73 ± 0.06	0.71 ± 0.08	
	MYLU	NA	NA	NA	0.69 ± 0.07	
	MYYU	NA	0.646 ± 0.16	0.53	0.68 ± 0.05	
InvSimpson	EPFU	5.16 ± 4.12	4.58 ± 3.42	3.86 ± 1.06	3.65 ± 1.10	
	MYLU	NA	NA	NA	3.42 ± 0.82	
	MYYU	NA	3.35 ± 1.82	2.15	3.41 ± 0.75	
Fisher’s	EPFU	12.60 ± 10.74	15.55 ± 11.53	14.72 ± 11.73	25.82 ± 20.39	
	MYLU	NA	NA	NA	9.73 ± 6.91	
	MYYU	NA	7.75 ± 4.24	5.22	9.89 ± 5.76	

^*a*^ EPFU, *Eptesicus fuscus*; MYYU, *Myotis yumanensis*; MYLU, *Myotis lucifugus*.

##### ITS data set

The alpha diversity of bat wing mycobiome was calculated after rarefying reads at 2,000 reads. One-way ANOVA on alpha diversity indices revealed that bats from all field sites showed significant differences in their alpha diversity indices for ITS (*P* < 0.05) when using the field site as an independent variable and alpha diversity as response variables (Table 3) (one-way ANOVA with BH P-value correction on bats from all sites: observed *P* = 2.295e-09, Chao1 *P* = 2.295e-09, ACE *P* = 8.616e-08, Shannon *P* = 2.111e-06, Simpson *P* = 0.004015, InvSimpson *P* = 0.0002784, Fisher *P* = 4.182e-09). Among the four field sites, the Central Lillooet site had significantly higher observed and Chao1 diversity indices than the North, West, and South Lillooet sites. Similarly, the Shannon index of fungal community at the Central Lillooet site is higher than that at the North and West Lillooet sites (Shannon: Central vs North *P* = 0.0018, Central vs West *P* = 1.5e-05). Different from that of bacterial diversity, bat species was not a strong predictor for fungal richness and diversity. Only the Shannon index showed a higher value for fungal diversity on EPFU wing swabs than that from MYYU (*P* = 0.014).

**TABLE 3:** Fungal species richness and alpha diversity indices estimated based on ITS metabarcode sequencing

Diversity index	Bat species^a^	Field site				
		North Lillooet	South Lillooet	Central Lillooet	West Lillooet	
Observed	EPFU	8.00 ± 2.12	26.87 ± 12.38	111.37 ± 87.25	19.14 ± 8.57	
	MYLU	NA	NA	NA	12.80 ± 9.92	
	MYYU	NA	17.67 ± 10.97	222.05	53.02 ± 9.56	
Chao1	EPFU	10.03 ± 2.83	23.22 ± 10.01	233.12 ± 205	38.66 ± 22.89	
	MYLU	NA	NA	NA	15.30 ± 6.39	
	MYYU	NA	8.0 ± 2	15.04	11.73 ± 9.75	
ACE	EPFU	10.21 ± 3.13	2.33 ± 10.20	273.87 ± 246	39.79 ± 23.86	
	MYLU	NA	NA	NA	14.50 ± 5.85	
	MYYU	NA	8.36 ± 1.49	15.83	16.85 ± 9.67	
Shannon	EPFU	1.19 ± 0.32	2.33 ± 0.53	2.78 ± 0.52	2.14 ± 0.47	
	MYLU	NA	NA	NA	1.87 ± 0.34	
	MYYU	NA	1.08 ± 0.36	2.04	1.73 ± 0.62	
Simpson	EPFU	0.58 ± 0.15	0.84 ± 0.11	0.79 ± 0.12	0.76 ± 0.09	
	MYLU	NA	NA	NA	0.77 ± 0.08	
	MYYU	NA	0.53 ± 0.14	0.83	0.65 ± 0.16	
InvSimpson	EPFU	2.54 ± 0.92	8.39 ± 4.11	6.83 ± 4.84	5.00 ± 2.71	
	MYLU	NA	NA	NA	5.11 ± 2.10	
	MYYU	NA	2.26 ± 0.72	5.92	5.29 ± 3.80	
Fisher’s	EPFU	1.46 ± 0.38	4.27 ± 2.17	43.65 ± 38.18	7.23 ± 5.12	
	MYLU	NA	NA	NA	2.46 ± 1.00	
	MYYU	NA	1.19 ± 0.35	2.50	3.14 ± 2.01	

^*a*^ EPFU, *Eptesicus fuscus*; MYYU, *Myotis yumanensis*; MYLU, *Myotis lucifugus*.

We compared fungal diversity of EPFU wings among the four field sites. The result agreed with the above that field sites influenced fungal richness and alpha diversity. EPFU from the Central Lillooet site had a significantly higher observed OTU richness than those from the West and South Lillooet sites (observed: Central vs West *P* = 0.0119, Central vs South *P* = 0.0036). However, the Simpson, InvSimpson, and Fisher diversity indices at the Central Lillooet site were significantly higher than those at the South Lillooet site (Central vs South: Simpson *P* = 0.0083; InvSimpson *P* = 0.0017; Fisher *P* = 0.0052) but not at the West or North Lillooet sites.

By analyzing fungal alpha diversity indices among the three bat species at the West Lillooet site, we did not detect any statistically significant difference. Thus, different from that of bacterial diversity, bat species did not seem to be a strong predictor for fungal alpha diversity indices (File S1).

### Beta diversity

#### 16S rRNA data set

**Fig 8:**
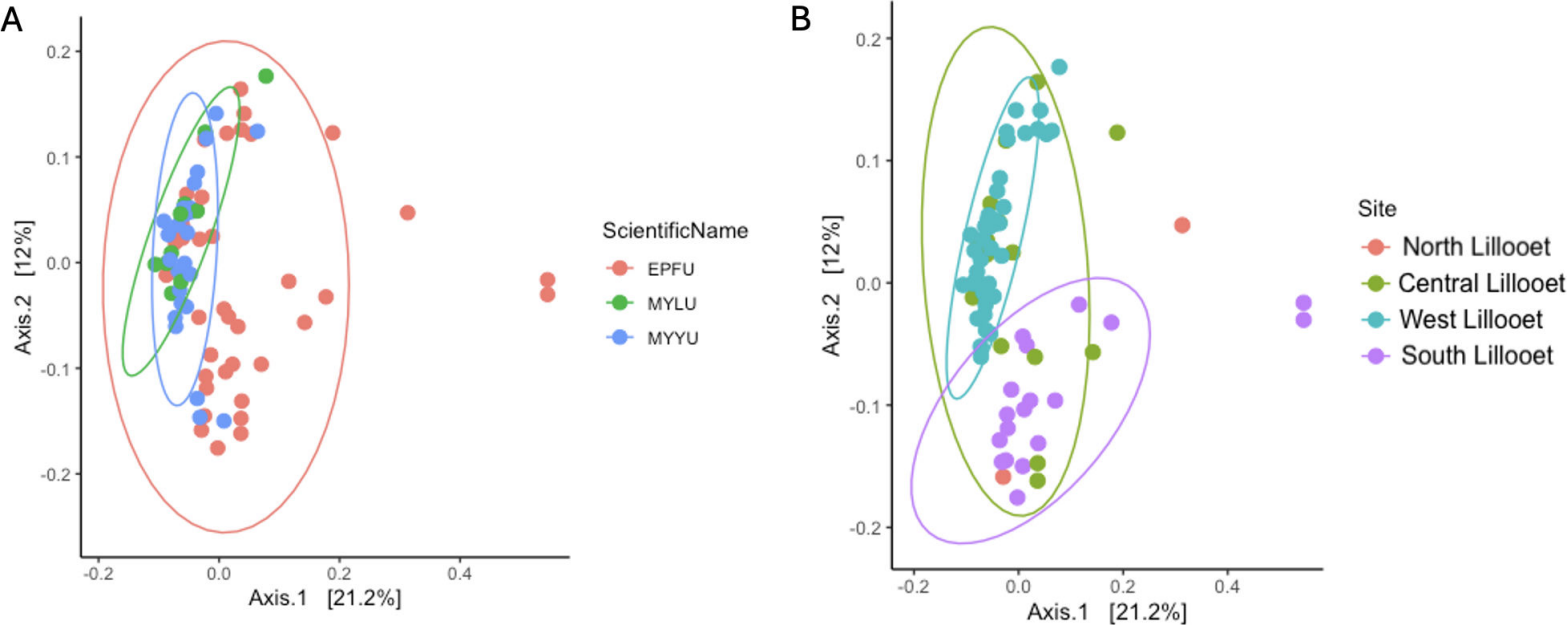
Principal coordinates analysis of Bray–Curtis distances among 76 bat wing bacterial communities. Fig. 5A highlighted the distance distributions within three bat species from all sites, while Fig. 5B highlighted those based on field sites.

We combined the wing bacterial communities of all 76 bats from the four field sites and performed principal coordinates analysis to visualize their relationships in a two-dimensional space (Fig. 8). Fig. 8A depicts the clustering pattern based on host bat species. Among the three bat species, EPFU showed a greater distribution range along both the first and second axes than the other two species. In contrast, MYYU and MYLU were more tightly clustered together, reflecting an overall high similarity of wing bacterial communities among bats from within and between these two species in Lillooet. Bat species was a weak but statistically significant predictor of beta diversity (*P* = 0.001, R^2^ = 0.006). Pairwise comparisons of EPFU vs MYYU (P.adj = 0.003) and EPFU vs MYLU showed significant differences between species pairs in beta diversity (P.adj = 0.036). However, no significant difference was observed between MYYU and MYLU (Fig. 8A).

Fig. 8B highlighted the clustering by field sites. Here, an overall significant difference was observed among the sites (*P* = 0.001, R^2^ = 0.164). Specifically, significant differences were found between the North vs West Lillooet sites (P.adj = 0.0210), the South vs Central Lillooet (P.adj = 0.0020) sites, the South Lillooet vs West Lillooet sites (P.adj = 0.0020), and the Central Lillooet vs West Lillooet sites (P.adj = 0.0020). The only comparison showing no significant difference in beta diversity was between the North Lillooet vs Central Lillooet sites (File S2).

**Fig 9:**
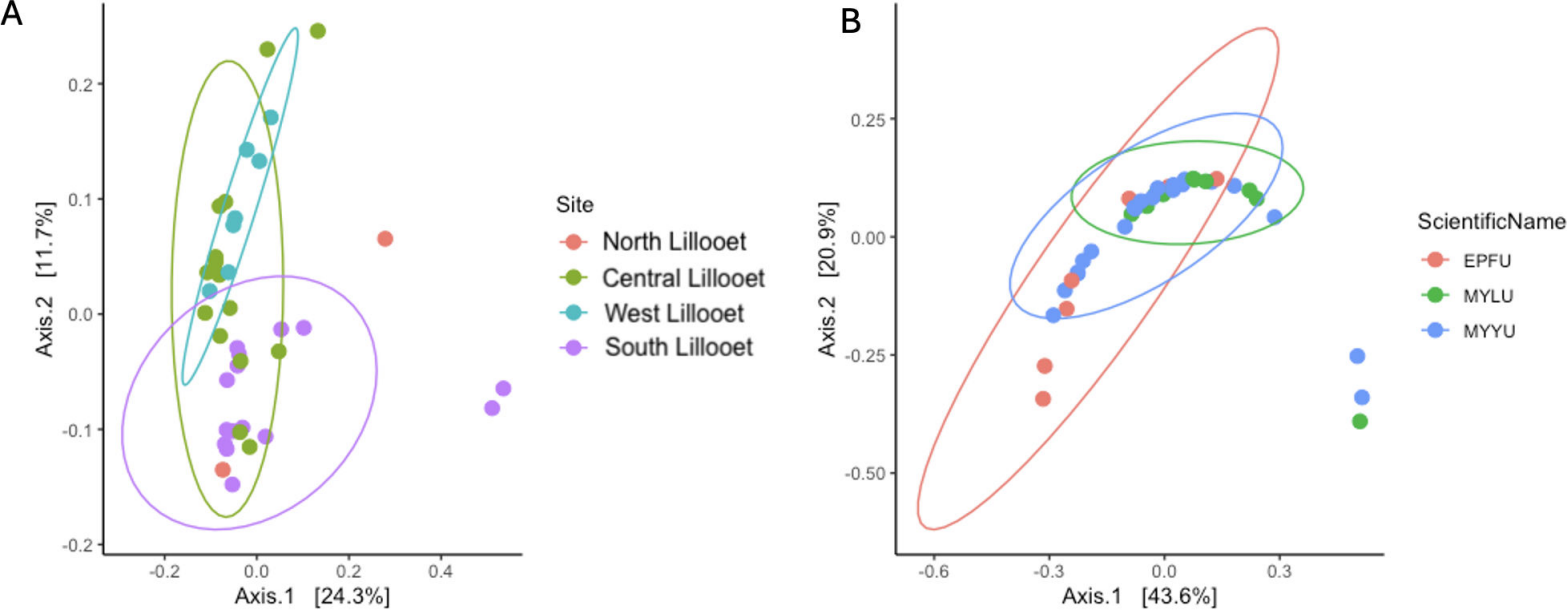
Principal coordinates analysis based on Bray–Curtis distances among bat wing bacterial communities. Fig. 6A highlighted the distance distributions among EPFU bats (*N* = 40) from all four field sites while Fig. 6B highlighted those (*N* = 39) within and among the three bat species within the West Lillooet site.

Because of the uneven distributions of sample sizes from different field sites and bat species and the potential confounding effects of these two factors when displaying the relationships, we further separately analyzed two subsets of data: (i) one species (EPFU) across all four sites and (ii) one site (West Lillooet) that contained all three bat species (Fig. 9). Our analyses revealed significant difference of bacterial beta diversity among the four sites for EPFU (*P* = 0.004, R^2^ = 0.167). Specifically, beta diversity of bacterial community on EPFU wings were significantly different between the South Lillooet vs Central Lillooet sites (P.adj = 0.0060), the South Lillooet vs West Lillooet sites (P.adj = 0.0320), and the Central Lillooet vs West Lillooet sites (P.adj = 0.0320) (Fig. 6A). At the West Lillooet site where we had samples from all the three bat species, our analysis showed an overall significant contribution of bat species to the observed bacterial diversity (*P* = 0.005, R^2^ = 0.099). Specifically, while MYYU vs MYLU comparison showed no statistically significant difference, EPFU vs MYYU (P.adj = 0.009) and EPFU vs MYLU (P.adj = 0.018) comparisons were significantly different from each other (Fig. 9B; File S2).

#### ITS data set

**Fig 10:**
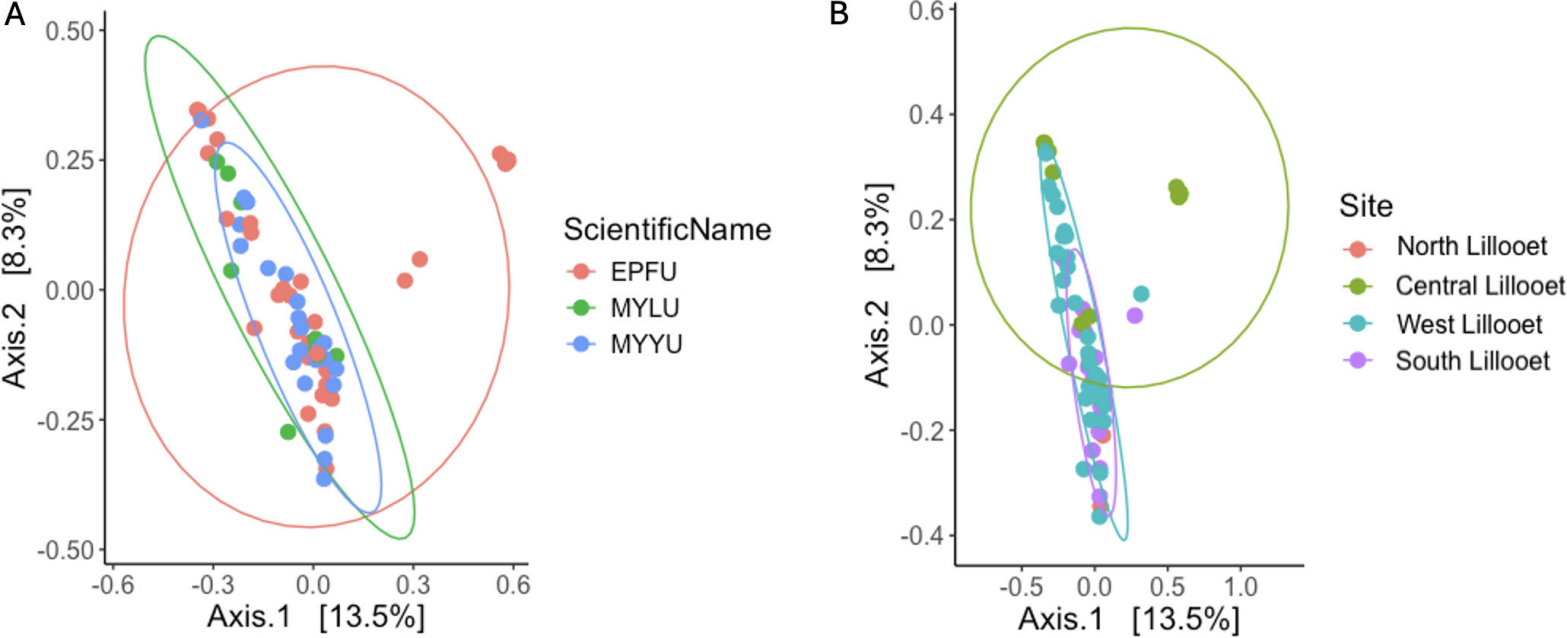
Principal coordinates analysis of Bray–Curtis distances among 76 bat wing fungal communities. Fig. 10A highlights the distance distributions within three bat species from all sites, while Fig. 10B highlights those based on four field sites.

**Fig 11:**
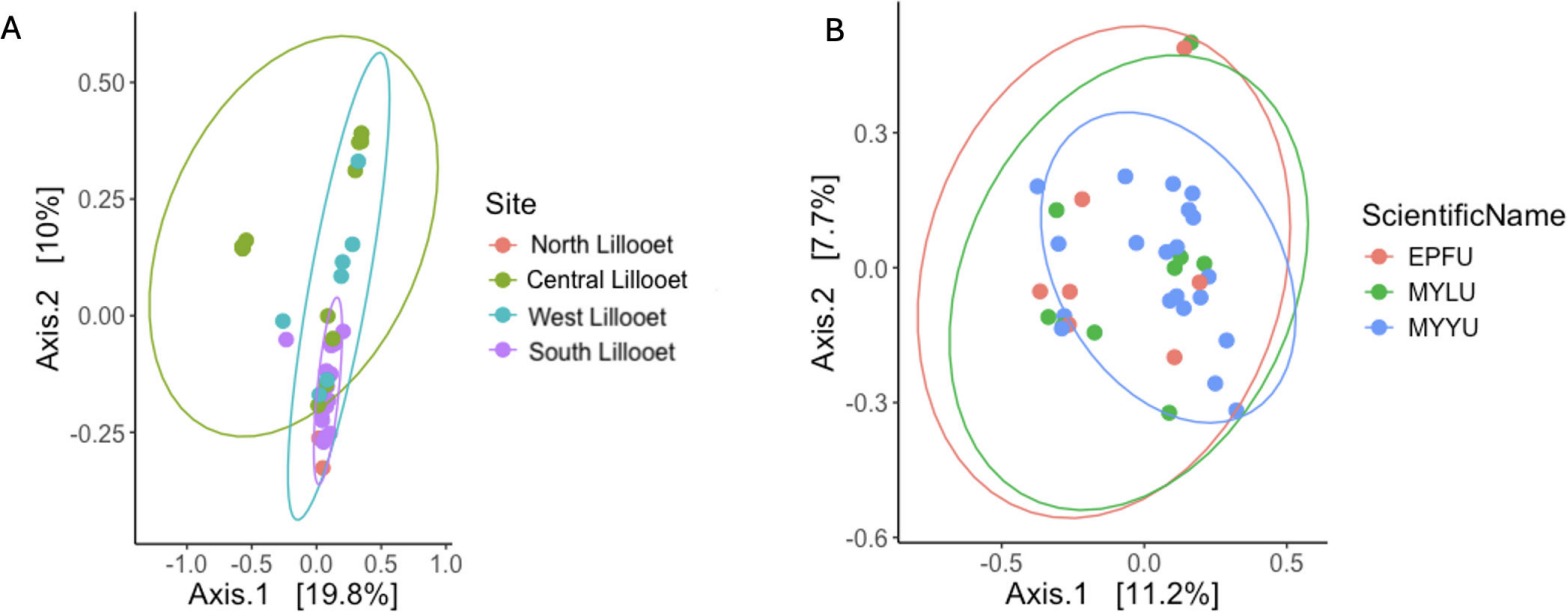
Principal coordinates analysis based on Bray–Curtis distances among bat wing fungal communities. Fig. 11A highlights the distance distributions among EPFU bats (*N* = 40) from all four field sites, while Fig. 11B highlights those (*N* = 39) within and among the three bat species within the West Lillooet site.

Principal coordinates analysis (PCoA) of the Bray–Curtis distance matrix shows the similarities in mycobiome compositions among all the 76 samples (Fig. 10, Fig. 11). Fig. 10 depicts the clustering pattern based on host bat species. Similar to those based on the 16S rRNA data set, among the three bat species, EPFU showed the greatest distribution range along the x-axis. MYYU and MYLU were clustered together (Fig. 10A). However, bat species was not a strong predictor of the beta diversity. No significant difference was observed between beta diversity among the three bat species (*P* = 0.053, R^2^ = 0.039). Fig. 10B highlighted the mycobiome clustering by field site. Here, significant differences in beta diversity were observed among all the sites (*P* = 0.001, R^2^ = 0.106), *e.g.*, North Lillooet vs Central Lillooet (P.adj = 0.008), North Lillooet vs West Lillooet (P.adj = 0.048), South Lillooet vs Central Lillooet (P.adj = 0.0045), South Lillooet vs West Lillooet (P.adj = 0.0045), and Central Lillooet vs West Lillooet (P.adj = 0.0045) (File S2).

In the same way as in 16S rRNA analysis, we mitigated the confounding effects that might come from uneven sample sizes and bat species by analyzing the two sub-data sets: EPFU from all four sites (Fig. 11A), and all three bat species at the West Lillooet site (Fig. 11B). Our analyses revealed significant differences of fungal diversity among the four field sites (*P* = 0.001, R^2^ = 0.155). Beta diversity of EPFU was significantly different among the four field sites. Significant differences were observed between the North vs South Lillooet sites (P.adj = 0.007), the North vs Central Lillooet sites (P.adj = 0.007), the North vs West Lillooet sites (P.adj = 0.007), the South vs Central Lillooet sites (P.adj = 0.004), the South vs West Lillooet sites (P.adj = 0.004), and the Central vs West Lillooet sites (P.adj = 0.004) (File S2). At the West Lillooet site where all three bat species were captured for analyses, our analyses showed no significant contribution of bat species to the observed fungal diversity (*P* = 0.699, R^2^ = 0.060), consistent with the result when performing clustering of bats from all four sites based on host bat species. Specifically, none of the three bat species at the West Lillooet site differed significantly from each other in their wing mycobiomes (Fig. 11B).

### Correlation between bacterial and fungal communities

We performed the Spearman rank correlation test from the ASVs abundance table to determine the potential relationship between fungal and bacterial richness/diversity. From all the alpha diversity indices used, we observed that among the 76 bat wing swab samples, bacterial richness/diversity was overall positively correlated with fungal richness/diversity (Table 4). Except for the inverse Simpson index, all other indices showed statistically significant positive correlation between bacterial and fungal diversities (*P* < 0.05).

**TABLE 4:** Spearman correlation between bacterial and fungal alpha diversity indices among the 76 bat wing microbiomes

Data		Spearman’s rho	S	*P*-value
16S rRNA	ITS			
Observed	Observed	0.315	44,373	0.006
Chao1	Chao1	0.307	44,912	0.008
ACE	ACE	0.353	36,980	0.003
Shannon	Shannon	0.259	48,046	0.027
InvSimpson	InvSimpson	0.16	54,442	0.175
Fisher’s	Fisher’s	0.315	44,373	0.006

Additionally, we investigated whether the relative abundances of several bacterial species (*Pseudomonas antarctica, Bacillus circulans,* and *Delftia tsuruhatensis*) were associated with the relative abundance of the fungal genus *Pseudogymnoascus* (which taxonomically contains the WNS pathogen *Pd*) on bat wings. Specifically, strains of *P. antarctica* and *B. circulans* have shown antagonistic activities against *Pseudogymnoascus* (34). In addition, *Delftia tsuruhatensis* was the most abundant bacterium on all 76 bat wings, and we sought to determine whether the relative abundance of this taxon was negatively correlated with that of *Pseudogymnoascus*. A significant negative correlation could suggest a potentially antagonistic interaction between *D. tsuruhatensis* and *Pd* on bats in western Canada, and with *D. tsuruhatensis* as a putative probiotic agent against *Pd*. However, our analyses revealed that none of the three correlation tests were statistically significant (File S3).

## DISCUSSION

In this study, we investigated bacterial and fungal communities on the wings of 76 bats belonging to three species collected at four field sites during a 5-day period in the summer in interior BC, western Canada. The four field sites were located within 40 km proximity of each other but differed in habitat type, with two sites being open grasslands, one site being partially forested grassland, and the fourth site was mountainous treed low-elevation riparian.

Information about bat wing bacterial and fungal communities were derived using the partial sequences of the 16S rRNA gene (for bacteria) and the ITS region (for fungi). Our analyses revealed extensive diversities of both bacteria and fungi on the wings of each bat. Interestingly, despite their relatively short geographic distances between field sites, bats from different sites often showed different microbial communities on their wings. In addition, within the same site, the bat species showed different relationships in their microbial communities. For example, the MYLU and MYYU had more similar wing microbiomes to each other than either were to EPFU. Overall, the wing microbiome from bats in Lillooet, BC, had different microbial communities from those from eastern and central Canada. Below, we compare those differences and discuss the implications of our observations to the managements of WNS in western Canada.

### Bacterial communities on bat wings

The prevalence of Proteobacteria in Lillooet is higher than those found in other parts of North America. For example, the proportions of Proteobacteria on individual bats in Ontario ranged from 24%–51%, with Actinobacteria (23%–53%) and Bacteroidetes (6%–38%) being similarly frequent as Proteobacteria (33) (File S4). However, sampling for that study (33) was performed during winter and thus aside from geographic differences, the temporal differences between our study and (33) could have contributed to the observed differences in bacterial taxa distributions. At the genus level, Lillooet bat wings were dominated by *Delftia*, *Phyllobacterium*, *Bordetella*, *Ralstonia*, *Sphingomonas*, *Bosea*, *Bradyrhizobium*, and *Pseudomonas*. Among the observed bacterial genera in relative high abundance in all bat species and sites, several are known as common members of the microbiomes of the plant phyllosphere, rhizosphere, and endosphere, including genera *Ralstonia*, *Phyllobacterium*, and *Bradyrhizobium* (*e.g.*, 61–63). *Delftia* was the most frequent bacterial genus in 75 of the 76 bats in Lillooet, ranging from 25% to 65%, with only one big brown bat at the Central Lillooet site having more *Pseudomonas* than *Delftia*. In contrast, the dominant bacterial genera in eastern and central Canada were *Arthrobacter*, *Chryseobacterium*, *Flavobacterium*, *Pedobacter*, *Mycoplana*, *Ralstonia*, and *Rhodococcus*, with genus *Ralstonia* being the most frequently shared between western and central/eastern Canada (33) (File S4). Avena et al. (64) studied wing microbiome of 11 bat species in eastern United States and Colorado. The phylum Proteobacteria was observed at high abundance, representing >65%, >52%, and >74% of the skin bacterial community of MYLU, MYYU, and EPFU, respectively. On their bat wings, the top bacterial classes for all samples were Gammaproteobacteria, Alphaproteobacteria, Actinobacteria, Betaproteobacteria, Bacilli, Flavobacteria, Cytophagia, and Thermoleophilia. Here, the top classes in our Lillooet bat wing microbiome were Gammaproteobacteria, Alphaproteobacteria, Actinobacteria, Bacilli, Bacteroidia, Clostridia, Cyanobacteria, Rubrobacteria, and Thermoleoplilia. While most of the abundant bacterial classes in the study of (64) were similar to those in our study, the high abundance of Bacilli, Bacteroidia and Clostridia seemed to be unique in the bat wing microbiome in Lillooet bats.

Previous studies showed that bat skin can be an excellent source of novel Actinobacteria and novel natural products that could benefit pathogen defense (65, 66). In one study, based on multilocus sequence analysis, 15 novel *Streptomyces* spp. were found on bat skin (65). In another study, 46% of actinobacterial isolates from bat skin exhibited ketosynthase (KS α) sequences with <85% sequence identity to known species and the dominant genera of Actinobacteria on bat wing skin were *Streptomyces* and *Micromonospora* (66). In addition, bat skin actinomycetes exhibited antifungal activity (88.9% of isolates). Potent antifungal producers that showed inhibition against *Pd* were in genera *Rhodococcus*, *Arthrobacter*, *Micrococcus*, *Streptosporangium*, *Luteipulveratus*, and *Nocardiopsis* (65). Actinobacteria are ubiquitous in soil and cave wall and bats likely to incorporate these environmental bacteria into their skin microbiome (64).

Among the four field sites around Lillooet, differences in the bat wing bacterial communities were also observed. However, the observed differences among the four field sites and the different bat species examined in Lillooet were less pronounced than the differences between the Lillooet bats and the eastern/central Canadian bats. Together, these results suggest that geographic separation and/or other factors could contribute to substantial bat wing microbiome differences. The study by Avena et al. (64) showed that geographic region was a stronger predictor of the variabilities of bat skin bacterial communities than bat species. Among the east/central Canada/USA regions versus the west Canadian regions including Lillooet, there are several differences, including climate, bat species ecology, and vegetation. Bats likely pick up their wing microbiome from their surrounding environments, including the air, cave walls, roost substrates, and their insect prey (64). Several eastern/central Canadian bats have been studied for their microbiomes, including EPFU (23), tricolored bat (32), and MYLU (23, 32, 35). Our study sites in Lillooet included two species (EPFU and MYLU) that overlapped with those reported in eastern/central Canada and revealed that intraspecific variation in bat wing microbiome between eastern/central Canada and Lillooet were greater than interspecific variation within each of the two regions. These two bat species differ greatly in their WNS susceptibility based on studies on eastern Canadian bats: MYLU has experienced substantial mortality from WNS and showed that the shifted skin microbiome was associated with the presence of *Pd* (32). On the other hand, EPFU has experienced lower WNS-caused mortality and less microbiome disruption than MYLU, consistent with the higher susceptibility of the latter species to WNS mortality (32).

We calculated bacterial diversity indices after we examined the rarefaction curves and rarefied data at 2,000 reads. At this read coverage, the observed bacterial species reached or approached the plateau of rarefaction curve in all samples, indicating that the depth of sequencing at this number of reads captured all or most bacterial diversity in each sample (67) (File S5). Among the three bat species that we sampled, the highest bacterial alpha diversity was seen in EPFU, particularly those from the West Lillooet site (*P* < 0.05); the highest fungal alpha diversity was also seen in EPFU, particularly those from the Central Lillooet site. These results are consistent with those of other studies that found greater diversity in wing microbiome of EPFU than of other bat species, such as MYYU (30), when considering all bat species and sites as main predictors for Shannon diversity index. Though they have overlapping preferences in their habitats, EPFU differs substantially from both MYYU and MYLU in their ecology. In contrast, MYLU and MYYU have similar ecologies to each other and have a high degree of overlap in their habitat selection including roosts and foraging areas (38). In our Lillooet study area, for example, both MYLU and MYYU prefer to forage immediately over calm water, and the largest number of captured bats was at the West Lillooet site, an area adjacent to Seton Lake, a large drinking and foraging feature. In contrast, EPFU bats feed higher above both aquatic and terrestrial features than MYYU and MYLU bats, approaching water only to drink (38), and the largest capture for this species was at the small fishponds at a bench high above the Fraser River (the Central Lillooet site). This site has several occupied bat boxes used by EPFU, and it is likely that many of the bats we sampled at this site were roost-mates. In response to daily weather changes or to disturbance, bats will move frequently among several roosts. When foraging or traveling, bats prefer to fly along habitat edges, with substantial activity concentrated in wetlands, riparian zones or within natural openings in forested areas. This use of multiple habitats can undoubtedly increase exposure to various microbes from different ecological niches, which bats can incorporate into the skin microbiome. Similarly, differences in diet between species could potentially influence the bat skin microbiome. However, the relationship between gut and skin microbiomes remains poorly understood.

Among the dominant bacteria that differ between Lillooet and eastern/central Canada and other places, *Delftia tsuruhatensis* stood out. This bacterium is a mesophilic, Gram-negative, terephthalate-assimilating motile bacterium in the class Gammaproteobacteria. *Delftia tsuruhatensis* was first isolated from activated sludge at a wastewater treatment plant in Japan (68) but has since been found in wastewater and other polluted environments in many areas of the world. Strains of *D. tsuruhatensis* have shown antimicrobial activity and have been hypothesized to suppress pathogens of plants through secondary metabolite production [summarized in (69)]. To the best of our knowledge, while microbes of the genus *Delftia* have been reported in bat ticks (70), the specific species *D. tsuruhatensis* has not been reported in bat microbiomes until our study. Interestingly, *D. tsuruhatensis* can be an opportunistic pathogen to immunocompromised humans, is known to be resistant to several antibiotics, is the subject of much study for environmental applications due to its ability to biodegrade organic contaminants, and is of medical interest due to its ability to produce an antimicrobial substance effective against numerous methicillin-resistant bacteria that cause human diseases, including *Staphylococcus aureus* [summarized in (69)]. How most of the bats in Lillooet came to harbor this bacterium in their wing microbiome, and in such high abundance, is not known. One possibility is that the environment in Lillooet, including the air sample, is dominated by this bacterium, causing all bats in the region containing abundant DNA of this species on their wings. Similarly, it is not known whether the observed high abundance of *D. tsuruhatensis* on bat wings was unique to Lillooet bats or if *D. tsuruhatensis* is commonly distributed across broad geographic regions in western Canada. Broad environmental sampling and analyses of air, soil, and water across western Canada is needed in order to address these issues.

What role *D. tsuruhatensis* might play in bat health or disease resistance is unknown and needs further investigation. Among the 76 analyzed bats, none showed obvious disease symptoms. Thus, it is unlikely that *D. tsuruhatensis* is pathogenic to the Lillooet bats; however, histopathological investigations are warranted to determine whether *D. tsuruhatensis* is pathogenic to bats. On the other hand, the secondary metabolites produced by this bacterium may have a beneficial effect for bats. Interestingly, it was recently discovered that *D. tsuruhatensis* secretes harmane, a substance that can penetrate mosquito cuticle and kill malaria parasite and suppress its transmission (71). Whether this bacterium can suppress the growth and reproduction of the white-nose syndrome fungus, *P. destructans*, is not yet known, but is in the future direction for our study.

We found variable proportions of *Pseudomonas* spp. in the wing microbiome, ranging from 0% to 12%. Several studies have shown that some *Pseudomonas* spp. have a strong anti-*Pd* activity (26, 27, 34). *Pseudomonas* spp. can produce pyoverdine, a florescent siderophore with high affinity to iron and to help them outcompete *Pd* in access to iron and limit *Pd* growth (72). In addition, gas chromatography–mass spectrometry (GC‐MS) analysis identified several volatile organic compounds, such as octanoic acid, 3‐tert‐butyl‐4‐hydroxyanisole (isoprenol), and 3‐methyl‐3‐buten‐1‐ol (BHA) produced by *Pseudomonas* species, such as *Pseudomonas yamanorum*, *Pseudomonas brenneri*, and *Pseudomonas fragi*, can limit the growth of *Pd* (34). In our earlier study (26), we specifically identified a strain of *P. antarctica* as an anti-*Pd* bacterium cultured from British Columbia, and this bacterium is one of the four *Pseudomonas* strains in a probiotic cocktail that is being tested for use as a prophylaxis against WNS in southwestern BC (73, 74, 75).

### Fungal communities on bat wings

Our analyses revealed that at the phyla level, Ascomycota, was the dominant fungal phylum on many Lillooet bat wings. Among the 76 bats, the proportion of Ascomycota ASVs ranged from 24%–96%, with genera *Cladosporium* (4%–40%) and *Aspergillus* (4%–88%) being similarly frequent Ascomycota members on bat wings. For bats in Canada, DNA metabarcode-based mycobiome has not been investigated. However, in Australian bats, the proportion of Ascomycota ranged from 0.6% to 95.4%, with genera *Aspergillus* (95.4%), *Rhodotorula* (85.9%), and *Urocladium* (86.5%) being dominant depending on the specific bats (76). For bats in New Mexico and Arizona in southwest US, the dominant fungal phylum on bat wing was Ascomycota (90.1%), followed by Mortierellomycota (4.4%) and Basidiomycota (2.5%) (28), similar to what we found here in Lillooet bats. Different from (77), we observed phyla Mucoromycotina and/or Zoopagomycota in a few bats representing both EPFU and MYLU. Phylum Zygomycota was recently split into phyla Mucoromycotina and Zoopagomycota and together, these two phyla contain about 1,000 species, representing <1% of total described fungi. Some species in these two phyla are known human pathogens and potentially could be detrimental to bat health.

At the genus level, Lillooet bat wings were dominated by *Cladosporium*, *Fusarium*, *Pseudogymnoascus*, and *Mortierella*. Similar genera were found by culture-based method on hibernating bats in Illinois and Indiana in Midwest US (78). In addition, similar results were found by cultured-based studies in eastern Canada (79). This high similarity in dominant fungal genera among diverse geographic regions is surprising because our sampling occurred in the heat of summer in Lillooet, typically one of the hottest regions/locations in Canada in the summer (80), while those earlier studies were on samples mostly obtained during other seasons (78, 79). Together, these results suggest that these fungi are likely generalists on bat wings.

Among the fungal assemblages on bat wings, *Aspergillus* were commonly found. *Aspergillus* is one of the biggest fungal genera, comprising 339 species that inhabit different types of environment (81). For example, spores of *Aspergillus penicillioides* have a high tolerance to heat and high pressure and have the ability to germinate at the low water activity (82). *Aspergillus gracilis* is an obligate halophilic fungus, first isolated from a hypersaline man-made saltern in Thailand (83). Observing *A. gracilis* on Lillooet bat wings may indicate the hypersalinity state of the wings. At present, the physicochemical characteristics of bat wings are not well understood. Vanderwolf et al. (84) reported that several fungi from bat wings can inhibit *Pd* growth *in vitro* only under specific salinity and pH conditions, suggesting the microenvironment on wings can influence microbial interactions and potentially WNS susceptibility. One of the fungal isolates, *Aureobasidium pullulans* 46379–835-2LNA could effectively inhibit *Pd* on yeast morphology agar medium at pH 5.0 with 6% NaCl after 2  weeks of incubation at 7°C (84). Other *Aureobasidium* isolates did not prefer high salt concentration to grow but could grow at a pH range of 4.5–7 on yeast morphology agar medium. Interestingly, incubation media were found to have notable effects on the anti-*Pd* activity of the fungal isolates from bat wings. For example, different media, including Sabouraud dextrose agar and brain heart infusion agar (with and without 10% sheep blood), showed distinctive *Pd*-inhibition patterns among the *Aureobasidium* isolates (84).

In addition to filamentous fungi, yeasts were also observed in high abundance across all species at the West Lillooet site, with *Rhodotorula* sequences found at 12%–80% among the bat wings. *Rhodotorula* is a saprophytic yeast, is ubiquitous in the environment, and can cause animal and human infections (85). Surprisingly, we did not observe lipophilic yeast genus *Malassezia* on bat wings. *Malassezia vespertilionis* has been reported as a common yeast that made up to 14.6% of wing mycobiome of bats from eastern USA (84). On the other hand, our discovery of *Rhodotorula* in high abundance is more similar to the study of mycobiome of bats in southern Australia (76) where *R. mucigilanosa* was among the most common yeasts on the wings of Eastern bent-winged bats (*Miniopterus orianae oceanensis*) at 1.3%–2.4%, and southern bent-winged bat (*M. orianae bassanii*) at 0.7%–85.9% of the total mycobiomes. Other yeasts found in our samples include *Candida*, a genus containing opportunistic pathogens infecting primarily immunocompromised humans (86).

We observed high numbers of reads assigned to bacteria and fungi associated with plants, including plant pathogens. For example, microorganisms in genera *Alternaria*, *Fomitopsis, Mycosphaerella*, *Ralstonia*, *Phyllobacterium,* and *Bradyrhizobium* were broadly distributed among our bat wing swabs. Plant-associated bacteria and fungi found on bats also emphasize the close relationships between bats and plants as bats in many regions serve as pollinators and seed dispersers. While bats in Canada, including those in Lillooet, do not serve these functions, these plant-associated bacteria and fungi were likely obtained by the bats roosting in trees or from surface-gleaning while preying insects on plant materials (87).

We found sequences of *Pseudogymnoascus* in 21 of the 76 bats that we captured. *Pseudogymnoascus* spp. are common on cave substrate worldwide and on hibernating bats in North America (32, 33, 88, 89, 90, 91, 92). At present, the sequenced ITS region is insufficient to clearly discriminate among many species within this genus. Our result is similar to that of (92) that obtained a high number of *Pd*-related ITS sequences from bat hibernacula but with a lack of taxonomic resolution. *Pd* in BC was first reported in West Kootenay in April 2023 (93). As we have been monitoring bats for WNS in Canada, we performed qPCR using Intergenic Spacer (IGS) primers and probes that are more specific for *Pd* than ITS primers (60). We confirmed that our samples from Lillooet were negative for this fungal species (these tests were repeated by Animal Health Centre in B.C.; unpublished data, Dr. Glenna McGregor). As we clustered ASVs using dynamic thresholds from the UNITE database, a specific threshold is needed to optimize the characterization of specific species to avoid splitting ITS sequences of one species into multiple species or lumping sister species together. The difficulty in differentiating *Pseudogymnoascus* spp. stems from its low variability in ITS barcode sequences between *P. destructans* versus *P. verrocosus* (less than 1% difference), and *P. pannorum* versus *P. roseus* (less than 3% difference) (ncbi.nlm.nih.gov). *Pd* is a psychrophilic fungus that grows optimally at 10–15°C, with little growth above 20°C (94). In the summer, the soil surface and air temperatures in Lillooet are typically much higher than 20°C. In addition, *Pd* is not known to actively infect bats during summer in North America (95). Together, these results indicated that the *Pseudogymnoascus* spp. found on Lillooet bats were unlikely *Pd*. Additional research, including culture-based assays, is needed to identify the specific species of *Pseudogymnoascus* on Lillooet bat wings.

### Limitations and implications

Other than geography and bat species discussed above, the sex of all 76 individual bats was also recorded. Overall, we observed no clear sex-specific microbiome clustering for either the bacterial or the fungal communities (Fig. 4 and 7). While some small sex-based clusters were found, bats in most of these clusters also shared the same ecological niches, captured in the same geographic site, and/or belonged to the same species, making it difficult to distinguish the effects of sex from those of bat species and geography. An earlier study also revealed limited effect of sex on bat wing microbiome (29).

Overall, compared with bats of EPFU, those of MYYU and MYLU showed similar microbiomes in alpha and beta diversity across the four analyzed field sites. This result reflects the ecology and behavior as MYYU and MYLU tend to roost and/or hibernate together. They have very similar ecological niches in terms of habitat and diet and prefer to stay in dense forest and forage near water bodies (38). However, the biased capture numbers among sites for the three species prevented us from conducting more robust analyses to quantify the relative contributions of all factors to the variations in bacterial and fungal communities on bat wings. During the night of 27 July 2022, we made another attempt to capture more bats at the North Lillooet site but without any success (likely due to northern lights we experienced during the night). The microbial diversity and the differences identified among field sites, among bat species, and potentially between bat sexes, warrant longitudinal studies to determine patterns of microbiome variation throughout the year. Lillooet has very high temperature in the summer (*e.g.*, July 2022: average 36°C during the day, 13°C at night) and low temperature in winter (*e.g.*, December 2022: average −24°C during the day, −19°C at night) (80). Such temperature fluctuations are known to impact bat distributions but can also influence the microbiome on bats. In addition, examination of sites with different climates would be informative. Our study provides a snapshot of the wing microbiome, which can be more dynamic throughout the year. Indeed, some members of the wing microbiome are likely transient microbes. Longitudinal study will also help to identify transient/ permanent microbiome on bat skin over time.

**Fig 12:**
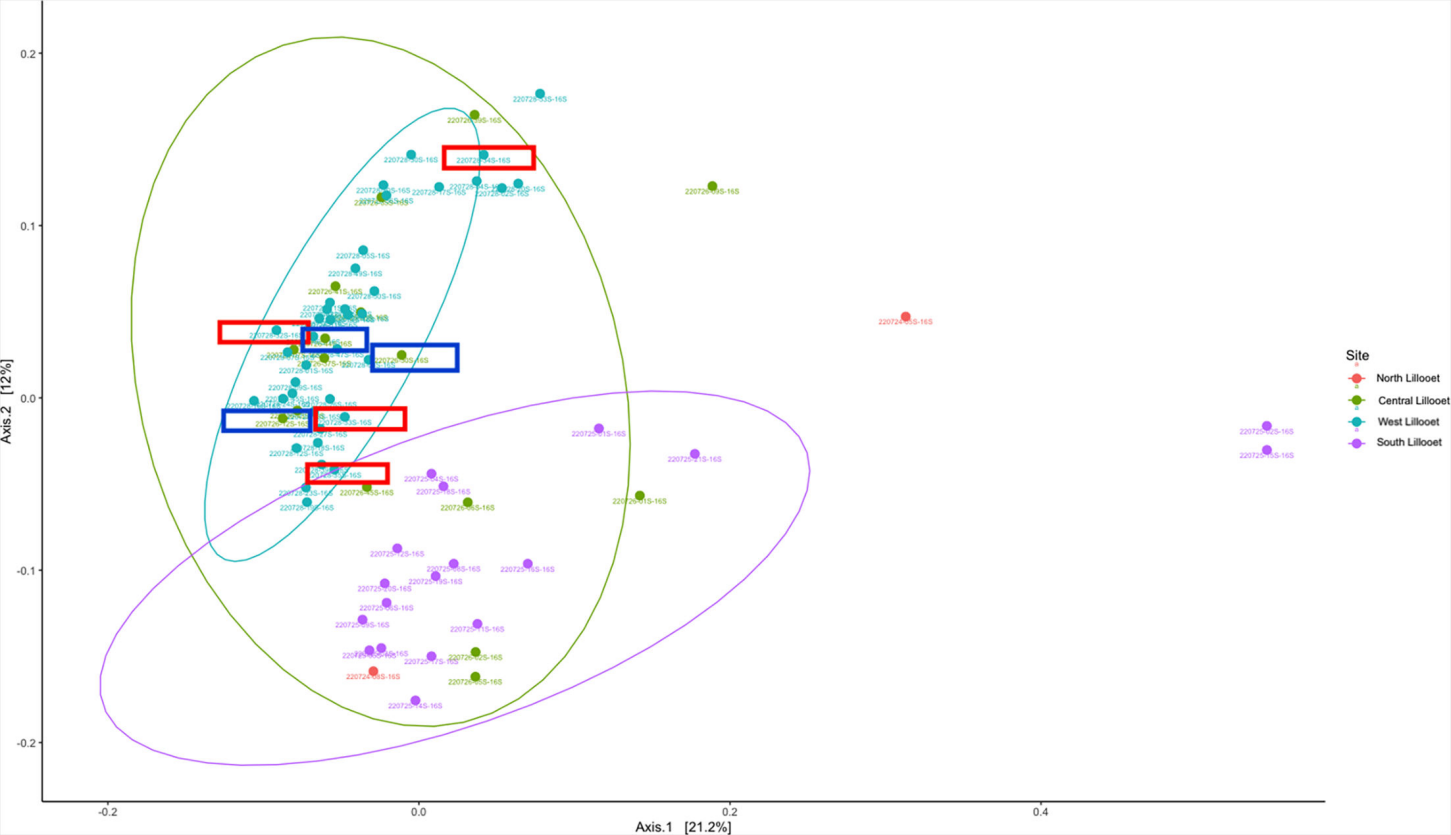
Principal coordinates analysis of Bray–Curtis distances among 76 bat wing bacterial communities showing time of bat capture and processing effects for six bats. Samples are colored based on field site. The six bats (two groups of three bats in each) captured and processed at around the same time are highlighted in the same colored rectangle boxes. Blue color represents bats captured and processed at around the same time during the night of 26 July 2022 at the South Lillooet site. Red color represents bats captured and processed around the same time during the night of 28 July 2022 at the West Lillooet site.

As mentioned earlier, the aerial microbial communities among the four sites might be different that could contribute to some of the observed differences among sites. To investigate this possibility, we identified two groups of bats that were captured and swabbed at around the same time (all within 10 min of each other) to see if their bacterial communities were clustered together on the PCoA plot. Two groups of bats each containing three bats were highlighted in Fig. 12. Our analyses revealed that the wing bacterial microbiomes within each group were not the closest to each other, consistent with the air microbiome not playing a major role. However, extensive air sampling and analyses throughout the bat capturing and processing are needed to quantify the potential contributions of aerial microbial communities at each site to the observed bat wing microbiome differences.

In this study, all the cloth bags used for keeping the bats calm and warm were cleaned, washed, and dried before their use. In addition, each bag was used at most only once each night. However, it is possible that the bags were contaminated by environmental microbes and/or microbial DNA. Such DNA, if existed, could potentially influence the wing microbiomes of bats kept within each bag. To investigate this possibility, we performed 16S rRNA metabarcoding analyses of wing swabs of two bats (a MYLU bat and a MYYU bat) captured at Colony Farm in Coquitlam, eastern Vancouver, BC in August 2022. These two bats were captured and processed using the same batch of bags, swabs, and microbial tubes, and followed the same methodology. The preliminary results are shown in File S6. A notable difference between these two samples and those from Lillooet was in the relative abundance of *Delftia*. Specifically, 4.49% (380 out of 22854 reads) of the 16S rRNA sequencing reads from the MYLU wing swab belonged to *Delftia*, while no *Delftia* sequence (0 out of 16226 reads) was found from the MYYU bat, both were much lower than those found on Lillooet bat wings (ranging from 25% to 65%). The results suggested that microbial DNA on cloth bags was unlikely to be a major contributor to the high abundance of *Delftia* bacteria on the wings of Lillooet bats. However, prior sampling and testing of these bags before their use are needed in order to quantify the potential contributions of microbial DNA on these bat holding bags to their wing microbiomes.

Bats in BC are not yet being impacted by *Pd* nor having any clinical signs of disease/ associated mass mortality*,* as opposed to those in eastern Canada and most of the US. The large differences in both bacterial and fungal communities identified between the Lillooet bats and those from eastern Canada suggest that prevention and treatment of WNS in western Canada may be different from those in eastern North America (77, 96, 97, 98, 99). Indeed, several microbes with anti-*Pd* activities have been found from western Canadian bats, some of these (*e.g.*, *Pseudomonas*) are found in our DNA metabarcode sequencing of the Lillooet bat wings. Notably, this anti-*Pd* wing bacterium was only found on EPFU, a species of bat that has shown lower WNS susceptibility over its eastern distribution (37). Based on previous studies, several abundant microbes that we identified on Lillooet bats using metabarcode sequencing have potential anti-*Pd* activity. Our study suggests that a potential approach for reducing impacts of WNS on western bats may entail developing and implementing strategies that enhance specific populations of microbes with anti-*Pd* activities on bat wings. From a basic microbiology perspective, the high number of unknown taxa from bat wings suggests that bat skin represents an underexplored source for novel microbiota.

### Conclusion

Our study revealed bacterial and fungal communities of bat wings in Lillooet, an area with among the highest bat diversity in Canada. Many bacterial and fungal species were found on most bat wings, including many potential novel species. Our analyses showed that both geographical location and host bat species contributed significantly to the diversity and distributions of bat wing microbiome in a relatively small region. Surprisingly, we observed that bat wings in Lillooet were highly enriched with *Delftia*, a rare or not-yet reported genus of bacteria on bats or in bat environments in other parts of North America. At present, the potential role of this bacterium to bat health is not known. In addition, we identified *Pseudomonas*, a genus of bacteria with anti-*Pd* activity (26), from the wing microbiome of many Lillooet bats. This study sheds light on the natural wing microbiome of Western Canadian bats and may help in planning biocontrol approach to protect bats from WNS.

## Data Availability

Data generated for this study can be found from Sequence Reads Archive (SRA) repository (https://www.ncbi.nlm.nih.gov/sra) under the BioProject PRJNA1027316

## SUPPLEMENTAL MATERIAL

Supplemental file 1 spectrum.00376-24-s0001.xlsx Bacterial and fungal diversity on bat wings.

Supplemental file 2 spectrum.00376-24-s0002.docx Comparisons of bacterial and fungal alpha diversities among sampling sites and bat species.

Supplemental file 3 spectrum.00376-24-s0003.docx Correlation tests between the relative abundance of *Pseudogymnoascus* and those of three selected bacterial species.

Supplemental file 4 spectrum.00376-24-s0004.docx Analysis of Lemieux-Labonte *et al*. (2017) data following our own pipeline.

Supplemental file 5 spectrum.00376-24-s0005.docx Rarefaction curves of bacterial and fungal metabarcoding among 76 bat wing swabs.

Supplemental file 6 spectrum.00376-24-s0006.xls Bacterial diversity on the wings of two bats from Colony Farm in Coquitlam, eastern Vancouver, BC, Canada.

ASM does not own the copyrights to Supplemental Material that may be linked to, or accessed through, an article. The authors have granted ASM a non-exclusive, world-wide license to publish the Supplemental Material files. Please contact the corresponding author directly for reuse.
