# Supplementary material for: DNA metabarcoding analyses reveal fine-scale microbiome structures on Western Canadian bat wings
Source: Microbiol Spectr. 2024 Oct 22;12(12):e00376-24. doi: 10.1128/spectrum.00376-24 (PMC11619579; doi:10.1128/spectrum.00376-24)
Supplement: Supplemental file 2 — Comparisons of bacterial and fungal alpha diversities among sampling sites and bat species. [file spectrum.00376-24-s0002.docx]

**Supplementary File 2**

**Comparisons of beta diversity among bat species and local sites in their bacterial and fungal communities**

**Bacterial diversity based on 16S rRNA metabarcode data**

1. All bats clustered by species


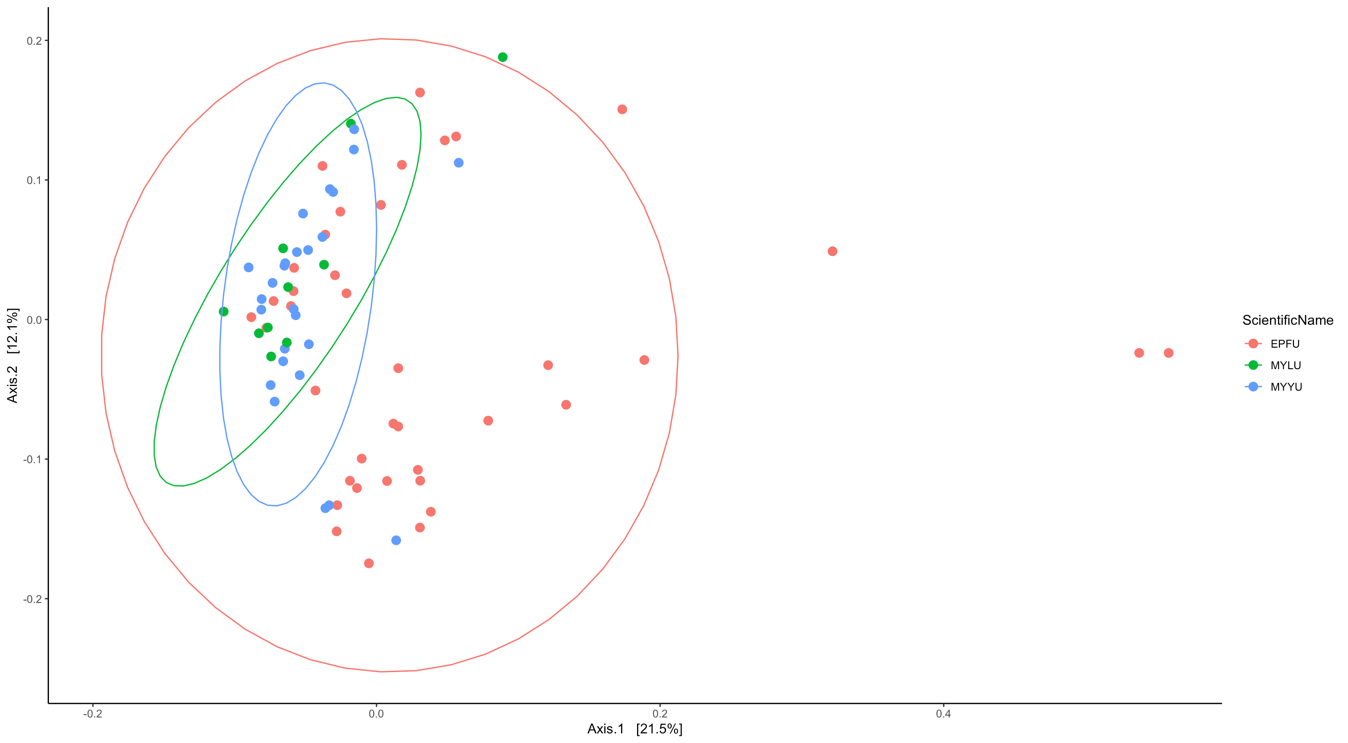


|  | Df | SumsOfSqs | MeanSqs | F.Model | R2 | Pr(>F) |
| --- | --- | --- | --- | --- | --- | --- |
| Bat species | 2 | 0.3045502 | 0.15227508 | 2.514439 | 0.06528562 | 0.001 |
| Residuals | 72 | 4.3603384 | 0.06056026 | NA | 0.93471438 | NA |
| Total | 74 | 4.6648886 | NA | NA | 1 | NA |

|  | 1 | 2 | p | p.adj |
| --- | --- | --- | --- | --- |
| 1 | EPFU | MYYU | 0.001 | 0.003 |
| 2 | EPFU | MYLU | 0.024 | 0.036 |
| 3 | MYYU | MYLU | 0.529 | 0.529 |

1. All bats clustered by field sites


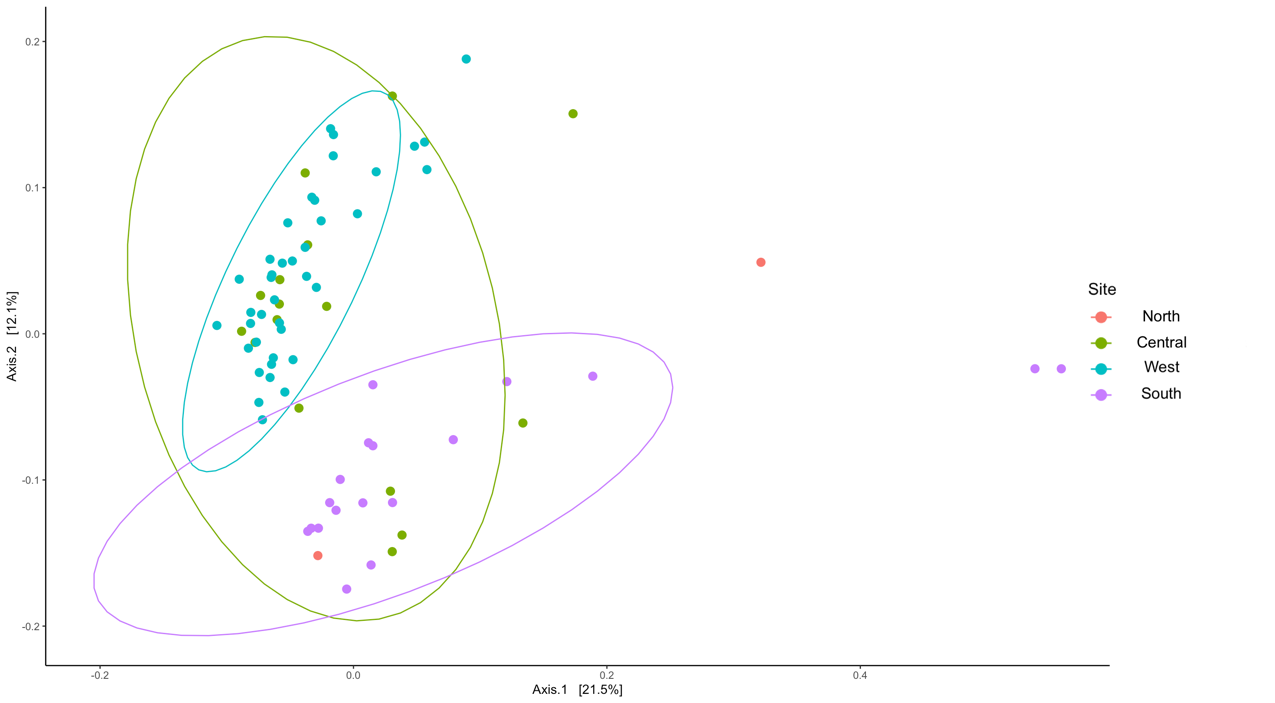


|  | Df | SumsOfSqs | MeanSqs | F.Model | R2 | Pr(>F) |
| --- | --- | --- | --- | --- | --- | --- |
| Site | 3 | 0.766339 | 0.25544634 | 4.652164 | 0.1642781 | 0.001 |
| Residuals | 71 | 3.89855 | 0.05490915 | NA | 0.8357219 | NA |
| Total | 74 | 4.664889 | NA | NA | 1 | NA |

|  | 1 | 2 | p | p.adj |
| --- | --- | --- | --- | --- |
| 1 | North Lillooet | South Lillooet | 0.23 | 0.23 |
| 2 | North Lillooet | Central Lillooet | 0.064 | 0.0768 |
| 3 | North Lillooet | West Lillooet | 0.014 | 0.021 |
| 4 | South Lillooet | Central Lillooet | 0.001 | 0.002 |
| 5 | South Lillooet | West Lillooet | 0.001 | 0.002 |
| 6 | Central Lillooet | West Lillooet | 0.001 | 0.002 |

1. EPFU at all four locations – effect of field site on wing microbiome


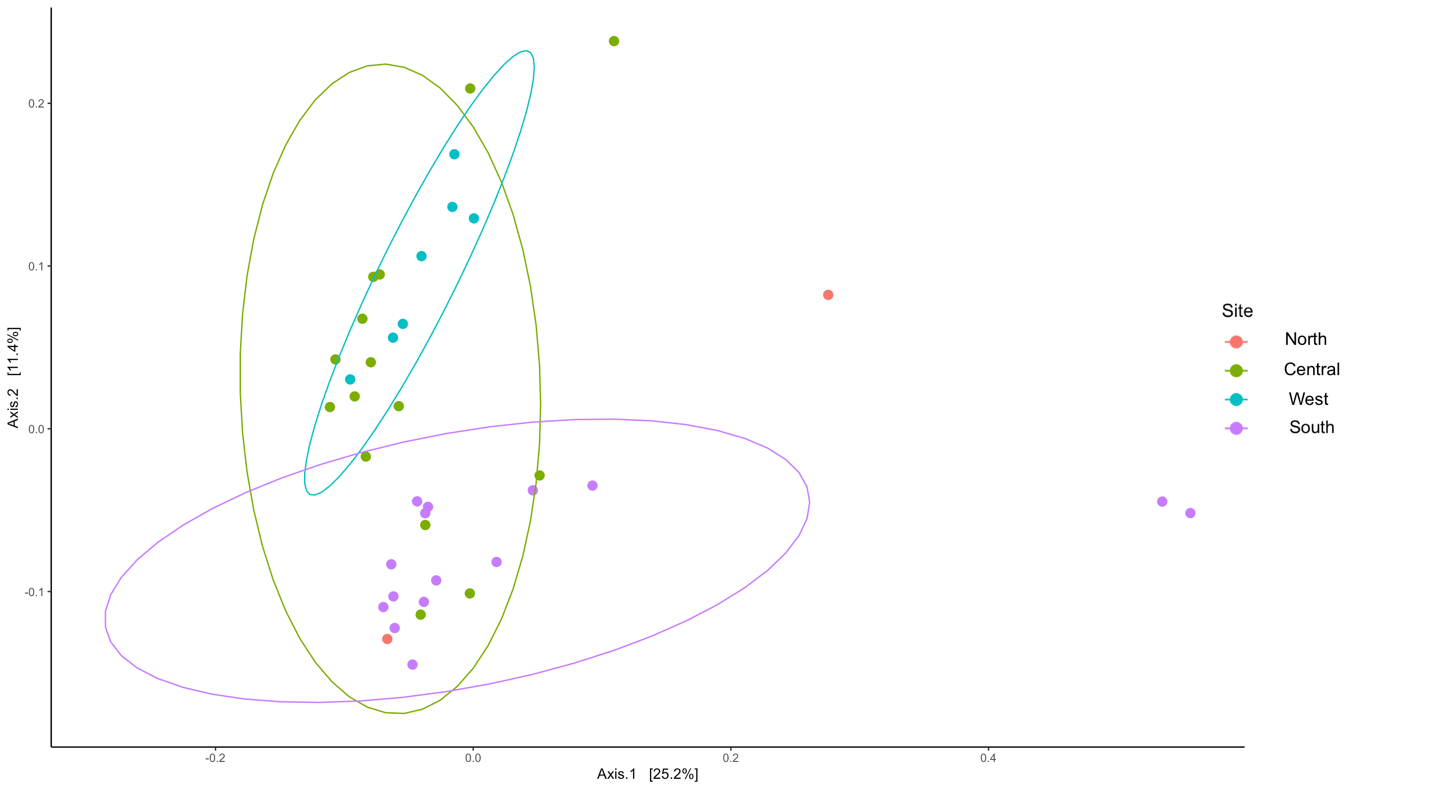


|  | Df | SumsOfSqs | MeanSqs | F.Model | R2 | Pr(>F) |
| --- | --- | --- | --- | --- | --- | --- |
| Site | 3 | 0.5439701 | 0.18132337 | 2.340795 | 0.1671106 | 0.004 |
| Residuals | 35 | 2.7111811 | 0.07746232 | NA | 0.8328894 | NA |
| Total | 38 | 3.2551512 | NA | NA | 1 | NA |

|  | 1 | 2 | p | p.adj |
| --- | --- | --- | --- | --- |
| 1 | North Lillooet | South Lillooet | 0.27 | 0.27 |
| 2 | North Lillooet | Central Lillooet | 0.063 | 0.0945 |
| 3 | North Lillooet | West Lillooet | 0.085 | 0.102 |
| 4 | South Lillooet | Central Lillooet | 0.001 | 0.006 |
| 5 | South Lillooet | West Lillooet | 0.016 | 0.032 |
| 6 | Central Lillooet | West Lillooet | 0.011 | 0.032 |

1. West Lillooet Site– all three bat species, effect of host species on wing microbiome


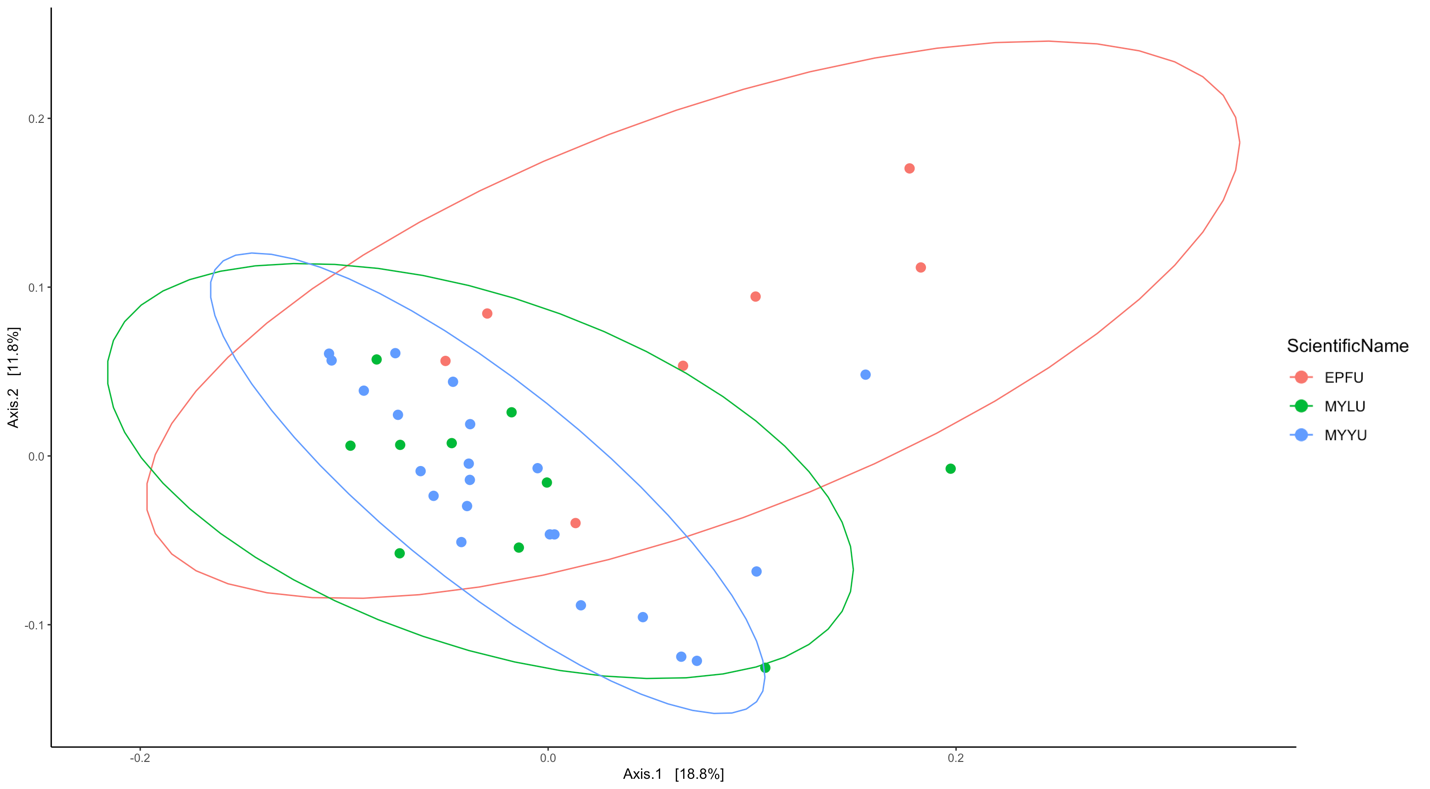


|  | Df | SumsOfSqs | MeanSqs | F.Model | R2 | Pr(>F) |
| --- | --- | --- | --- | --- | --- | --- |
| Bat species | 2 | 0.1510117 | 0.07550586 | 1.974644 | 0.09885755 | 0.005 |
| Residuals | 36 | 1.3765572 | 0.0382377 | NA | 0.90114245 | NA |
| Total | 38 | 1.5275689 | NA | NA | 1 | NA |

|  | 1 | 2 | p | p.adj |
| --- | --- | --- | --- | --- |
| 1 | EPFU | MYYU | 0.003 | 0.009 |
| 2 | EPFU | MYLU | 0.012 | 0.018 |
| 3 | MYYU | MYLU | 0.804 | 0.804 |

**Fungal diversity based on ITS metabarcode data**

1. All bats clustered by species


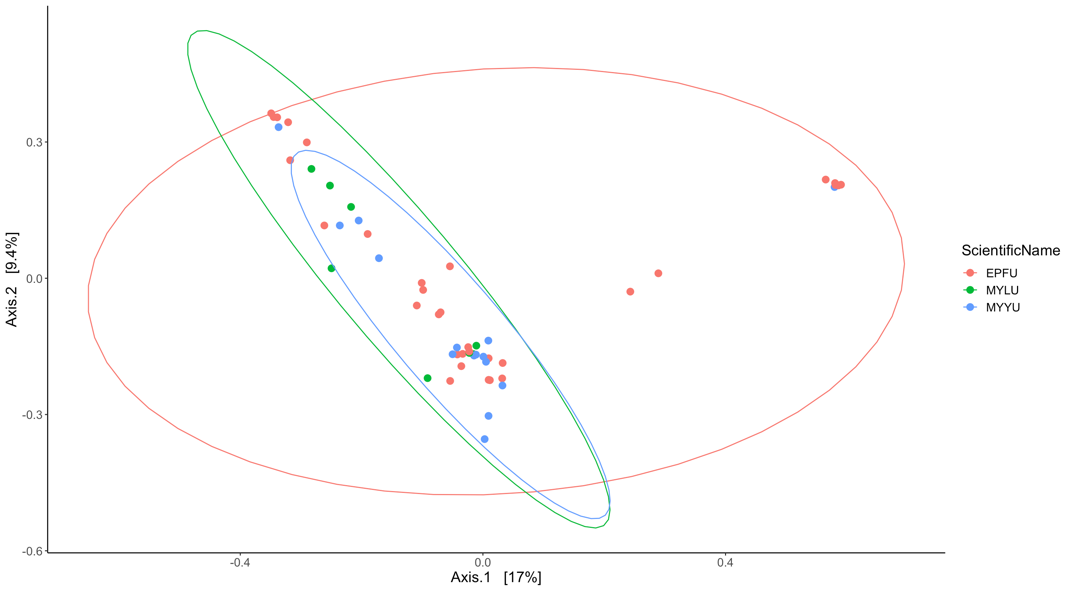


|  | Df | SumsOfSqs | MeanSqs | F.Model | R2 | Pr(>F) |
| --- | --- | --- | --- | --- | --- | --- |
| Bat species | 2 | 1.161346 | 0.580673 | 1.318632 | 0.03957643 | 0.053 |
| Residuals | 64 | 28.183041 | 0.44036 | NA | 0.96042357 | NA |
| Total | 66 | 29.344387 | NA | NA | 1 | NA |

|  | 1 | 2 | p | p.adj |
| --- | --- | --- | --- | --- |
| 1 | EPFU | MYYU | 0.049 | 0.147 |
| 2 | EPFU | MYLU | 0.174 | 0.261 |
| 3 | MYYU | MYLU | 0.828 | 0.828 |

1. All bats clustered by field sites


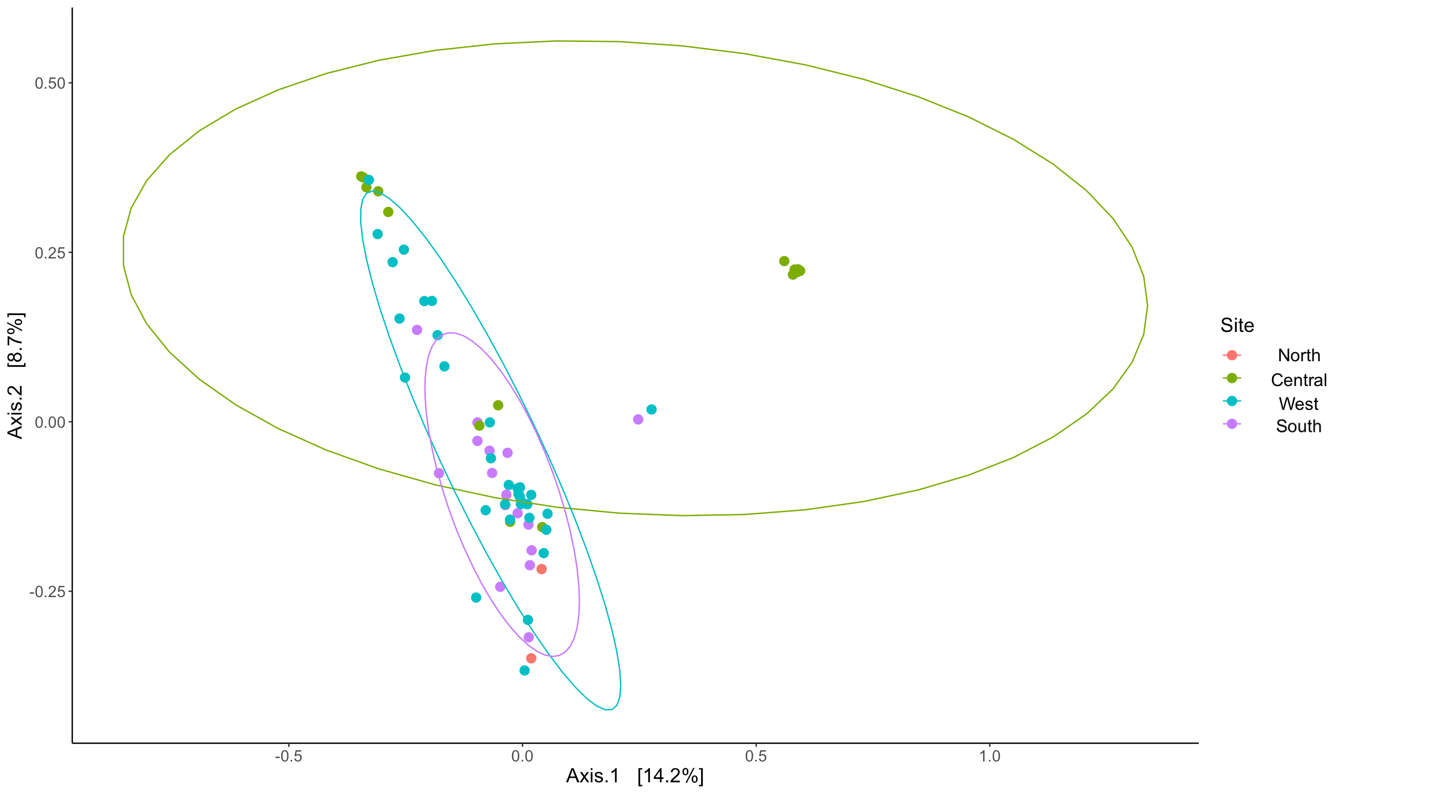


|  | Df | SumsOfSqs | MeanSqs | F.Model | R2 | Pr(>F) |
| --- | --- | --- | --- | --- | --- | --- |
| Site | 3 | 3.124001 | 1.0413338 | 2.502024 | 0.1064599 | 0.001 |
| Residuals | 63 | 26.220385 | 0.4161966 | NA | 0.8935401 | NA |
| Total | 66 | 29.344387 | NA | NA | 1 | NA |

|  | 1 | 2 | p | p.adj |
| --- | --- | --- | --- | --- |
| 1 | North Lillooet | South Lillooet | 0.003 | 0.0045 |
| 2 | North Lillooet | Central Lillooet | 0.008 | 0.008 |
| 3 | North Lillooet | West Lillooet | 0.004 | 0.0048 |
| 4 | South Lillooet | Central Lillooet | 0.002 | 0.0045 |
| 5 | South Lillooet | West Lillooet | 0.003 | 0.0045 |
| 6 | Central Lillooet | West Lillooet | 0.001 | 0.0045 |

1. EPFU at all four locations – effect of field site on wing mycobiome


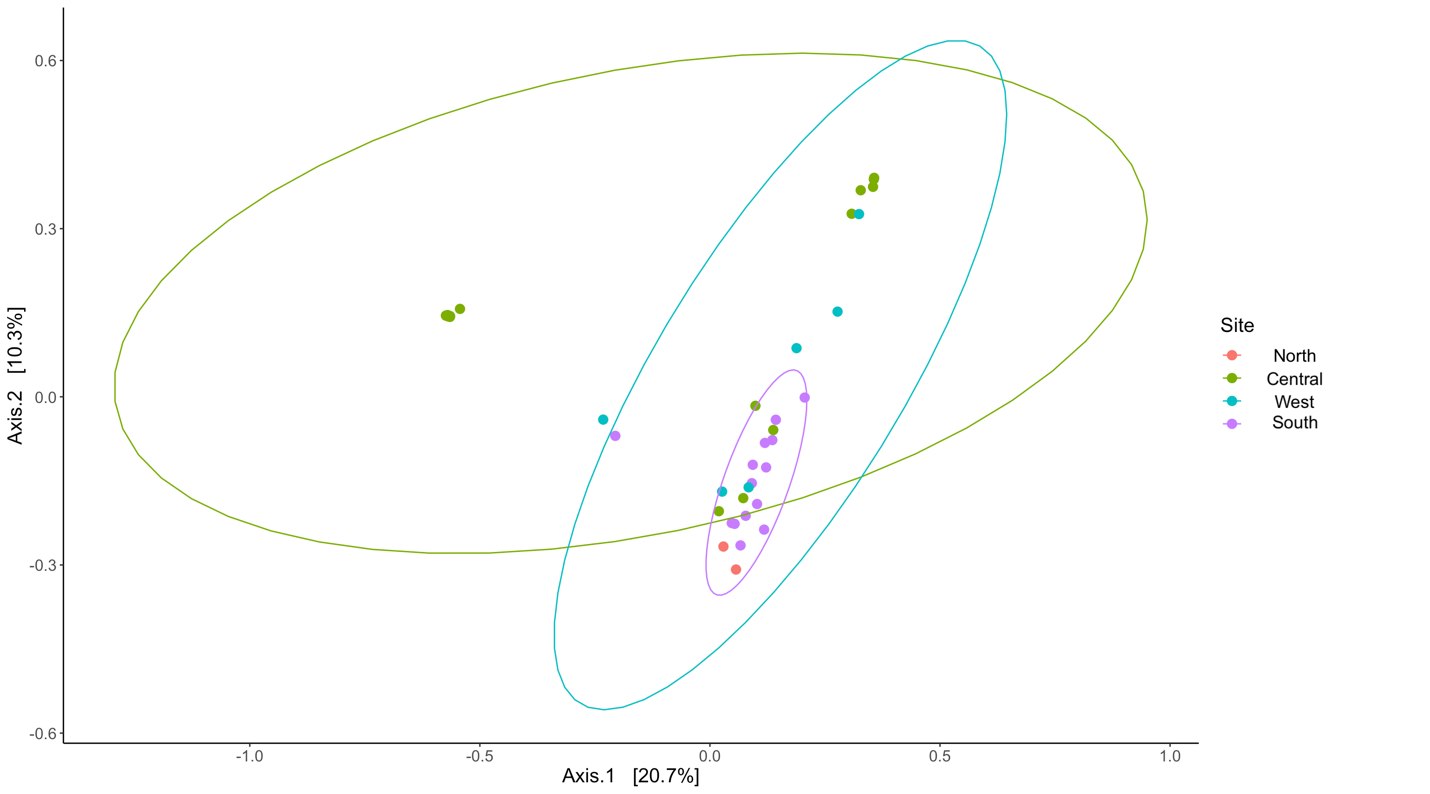


|  | Df | SumsOfSqs | MeanSqs | F.Model | R2 | Pr(>F) |
| --- | --- | --- | --- | --- | --- | --- |
| Site | 3 | 2.494296 | 0.8314321 | 2.072766 | 0.1546137 | 0.001 |
| Residuals | 34 | 13.638146 | 0.4011219 | NA | 0.8453863 | NA |
| Total | 37 | 16.132442 | NA | NA | 1 | NA |

|  | 1 | 2 | p | p.adj |
| --- | --- | --- | --- | --- |
| 1 | North Lillooet | South Lillooet | 0.007 | 0.007 |
| 2 | North Lillooet | Central Lillooet | 0.006 | 0.007 |
| 3 | North Lillooet | West Lillooet | 0.005 | 0.007 |
| 4 | South Lillooet | Central Lillooet | 0.002 | 0.004 |
| 5 | South Lillooet | West Lillooet | 0.002 | 0.004 |
| 6 | Central Lillooet | West Lillooet | 0.001 | 0.004 |

1. West Lillooet Site– all three bat species, effect of host species on wing mycobiome


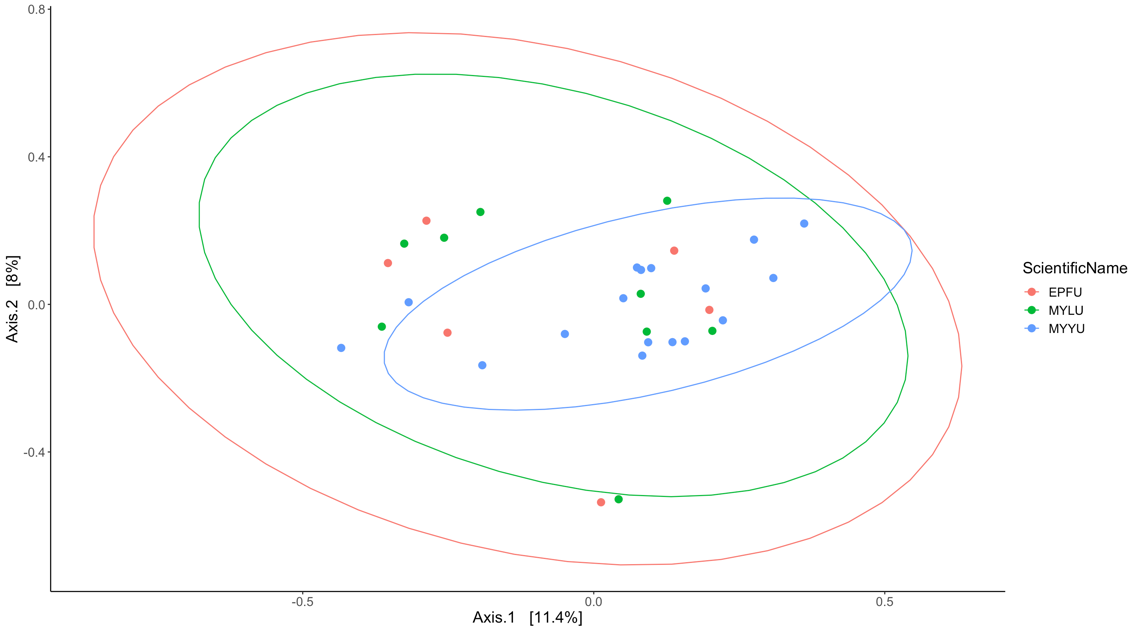


|  | Df | SumsOfSqs | MeanSqs | F.Model | R2 | Pr(>F) |
| --- | --- | --- | --- | --- | --- | --- |
| Bat species | 2 | 0.8111618 | 0.4055809 | 0.9197748 | 0.05964904 | 0.699 |
| Residuals | 29 | 12.7877462 | 0.4409568 | NA | 0.94035096 | NA |
| Total | 31 | 13.598908 | NA | NA | 1 | NA |
