# Supplementary material for: DNA metabarcoding analyses reveal fine-scale microbiome structures on Western Canadian bat wings
Source: Microbiol Spectr. 2024 Oct 22;12(12):e00376-24. doi: 10.1128/spectrum.00376-24 (PMC11619579; doi:10.1128/spectrum.00376-24)
Supplement: Supplemental file 3 — Correlation tests between the relative abundance of Pseudogymnoascus and those of three selected bacterial species. [file spectrum.00376-24-s0003.docx]

**Supplementary File 3**

**Correlation tests between the relative abundance of Pseudogymnoascus and those of three selected bacterial species**

| Data | | Spearman’s rho | S | P-value |
| --- | --- | --- | --- | --- |
| Fungus | Bacterium |  |  |  |
| *Pseudogymnoascus* | *Delftia tsuruhatensis* | -0.1103788 | 81224 | 0.3425 |
| *Pseudogymnoascus* | *Pseudomonas antarctica* | -0.1182324 | 81799 | 0.3091 |
| *Pseudogymnoascus* | *Bacillus circulans* | -0.01898147 | 74538 | 0.8707 |
