# Supplementary material for: DNA metabarcoding analyses reveal fine-scale microbiome structures on Western Canadian bat wings
Source: Microbiol Spectr. 2024 Oct 22;12(12):e00376-24. doi: 10.1128/spectrum.00376-24 (PMC11619579; doi:10.1128/spectrum.00376-24)
Supplement: Supplemental file 4 — Analysis of Lemieux-Labonte et al. (2017) data following our own pipeline. [file spectrum.00376-24-s0004.docx]

Supplementary File 4

Analysis of Lemieux-Labonte et al., (2017) data following our own pipeline. Raw sequences were retreived from figshare :

Raw sequence files: <https://figshare.com/s/623a1e47b4bed20459a7>

Metadata: <https://figshare.com/s/74d9497a792f9c0c76df>).

Demultiplexed sequences were processed using cutadapt for primer removal. DADA2 v.1.22 was used for sample inference and denoising to assign taxonomy to individual sequences because of its high accuracy and high resolution for inferring amplicon sequence variants (ASVs) with even 1 or 2 nucleotide differences. Paired end reads were truncated at decreasing quality scores and assembled. After quality filtering and taxonomic assignment, an abundance table was constructed. This abundance table records the number of sequence reads for each ASV. Taxonomy was assigned to representative sequences using naive-Bayes classifier trained against a reference database (16S rRNA: SILVA release 138 at 99%)


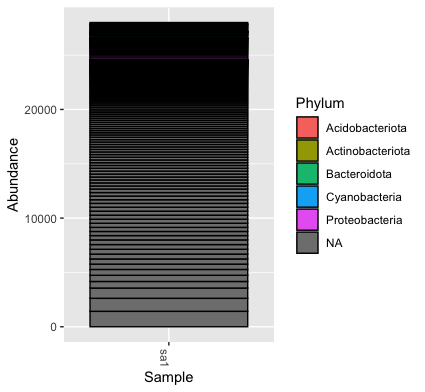


Fig 1. Relative abundance of 16S rRNA reads of bacteria at the phylum level.


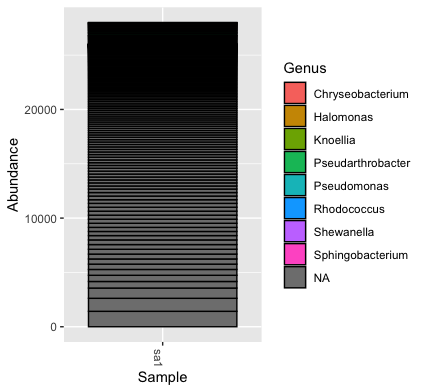


Fig 2. Relative abundance of 16S rRNA reads of bacteria at the genus level.


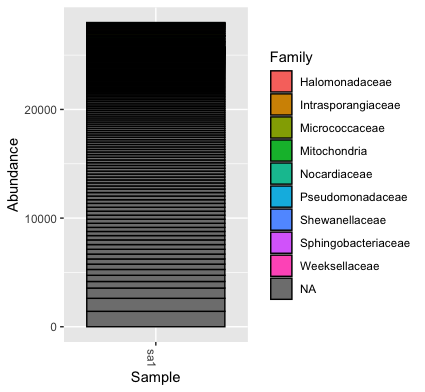


Fig 3. Relative abundance of 16S rRNA reads of bacteria at the family level.
