# Supplementary material for: DNA metabarcoding analyses reveal fine-scale microbiome structures on Western Canadian bat wings
Source: Microbiol Spectr. 2024 Oct 22;12(12):e00376-24. doi: 10.1128/spectrum.00376-24 (PMC11619579; doi:10.1128/spectrum.00376-24)

**Supplementary File 5**

**Rarefaction curves of bacterial and fungal metabarcoding among 76 bat wing swabs**

16S rRNA


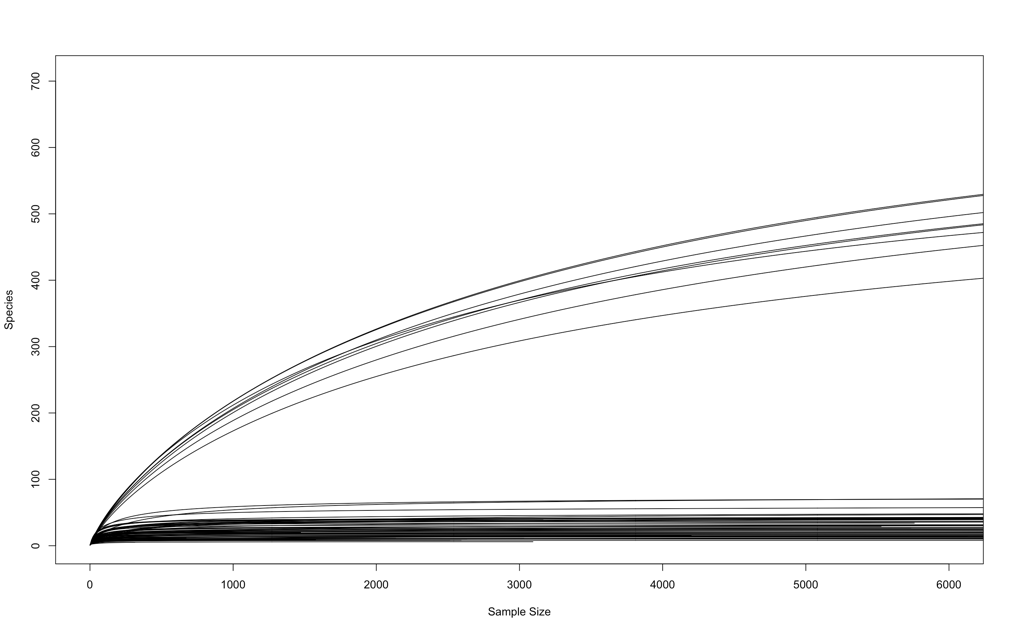


ITS


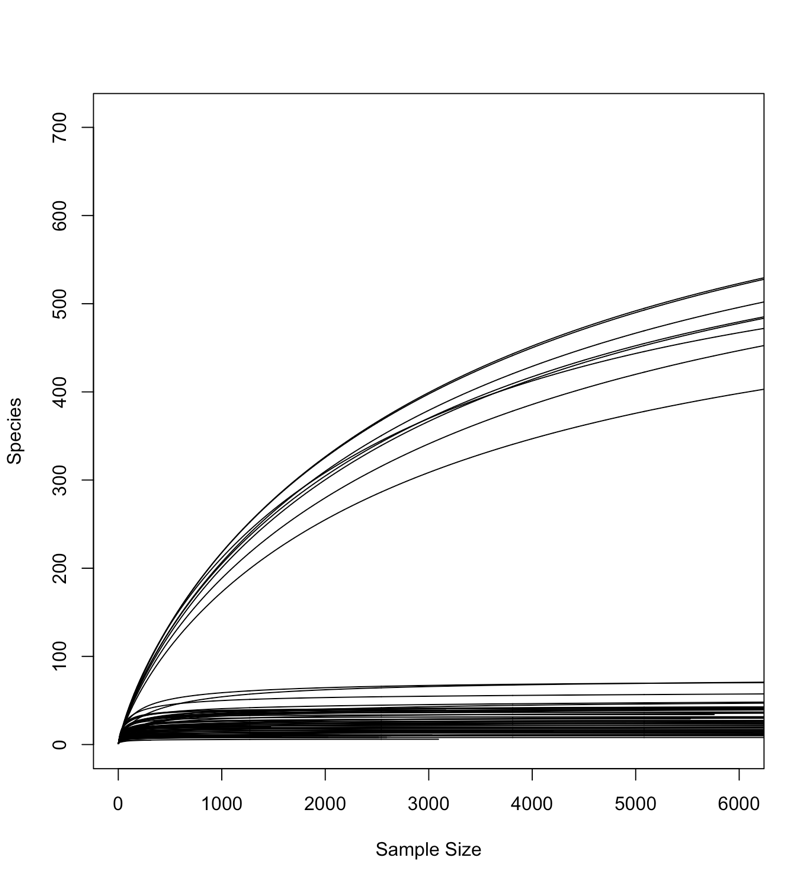

Supplement: Supplemental file 5 — Rarefaction curves of bacterial and fungal metabarcoding among 76 bat wing swabs. [file spectrum.00376-24-s0005.docx]
